# An overview of fish bioacoustics and the impacts of anthropogenic sounds on fishes†

**DOI:** 10.1111/jfb.13948

**Published:** 2019-04-05

**Authors:** Arthur N. Popper, Anthony D. Hawkins

**Affiliations:** ^1^ Department of Biology University of Maryland College Park Maryland USA; ^2^ Loughine Ltd. Aberdeen UK

**Keywords:** behaviour, criteria, effects, guidelines, hearing, sound

## Abstract

Fishes use a variety of sensory systems to learn about their environments and to communicate. Of the various senses, hearing plays a particularly important role for fishes in providing information, often from great distances, from all around these animals. This information is in all three spatial dimensions, often overcoming the limitations of other senses such as vision, touch, taste and smell. Sound is used for communication between fishes, mating behaviour, the detection of prey and predators, orientation and migration and habitat selection. Thus, anything that interferes with the ability of a fish to detect and respond to biologically relevant sounds can decrease survival and fitness of individuals and populations.

Since the onset of the Industrial Revolution, there has been a growing increase in the noise that humans put into the water. These anthropogenic sounds are from a wide range of sources that include shipping, sonars, construction activities (*e.g*., wind farms, harbours), trawling, dredging and exploration for oil and gas. Anthropogenic sounds may be sufficiently intense to result in death or mortal injury. However, anthropogenic sounds at lower levels may result in temporary hearing impairment, physiological changes including stress effects, changes in behaviour or the masking of biologically important sounds.

The intent of this paper is to review the potential effects of anthropogenic sounds upon fishes, the potential consequences for populations and ecosystems and the need to develop sound exposure criteria and relevant regulations. However, assuming that many readers may not have a background in fish bioacoustics, the paper first provides information on underwater acoustics, with a focus on introducing the very important concept of particle motion, the primary acoustic stimulus for all fishes, including elasmobranchs. The paper then provides background material on fish hearing, sound production and acoustic behaviour. This is followed by an overview of what is known about effects of anthropogenic sounds on fishes and considers the current guidelines and criteria being used world‐wide to assess potential effects on fishes.

Most importantly, the paper provides the most complete summary of the effects of anthropogenic noise on fishes to date. It is also made clear that there are currently so many information gaps that it is almost impossible to reach clear conclusions on the nature and levels of anthropogenic sounds that have potential to cause changes in animal behaviour, or even result in physical harm. Further research is required on the responses of a range of fish species to different sound sources, under different conditions. There is a need both to examine the immediate effects of sound exposure and the longer‐term effects, in terms of fitness and likely impacts upon populations.

## 1 INTRODUCTION

The past several decades have seen an increasing level of interest in the potential effects of anthropogenic sounds on aquatic life. The sounds added by humans into aquatic environments (both marine and freshwater), include those from shipping, dredging, sonars, seismic airguns used for oil and gas exploration, underwater explosions and construction, including pile driving, as well as many other activities. Anthropogenic sounds such as these have increased in recent times as a result of increases in shipping, harbour developments, the construction and operation of offshore wind farms, tidal and wave energy generation, dredging and cable and pipe laying, seismic surveys for oil and gas and offshore oil developments. Although initial concern regarding anthropogenic sound focussed on the effects upon marine mammals (NMFS, 2018; NRC, 1994; Southall *et al.,* 2007), there is now growing concern over potential effects upon those organisms that make up a much larger part of the aquatic biomass, fishes and more recently, invertebrates and zooplankton (Popper & Hawkins, 2016). Concern has also been expressed recently over effects upon freshwater fishes (Bolgan *et al.,* 2016; Mickle & Higgs, 2018), since they have received far less attention in research on noise effects studies.

The added sounds in the aquatic environment may have a wide range of effects on fishes. Exposure to very intense sounds may result in mortal injuries, but far more important issues are associated with sounds that are detectable by fishes and which may affect their behaviour, causing them to move away from their migration routes, leave favoured habitats in which they feed or breed, interfere with communication using sound, affect reproductive behaviour (where sound is used to attract mates and facilitate spawning), or prevent the detection of other biologically important sounds. As a consequence, the addition of anthropogenic sounds to the aquatic environment has the potential to do significant harm to fishes.

From an historical perspective, fish bioacoustic studies up until the early 21st century asked basic questions about hearing, sound communication and behaviour. While such studies continue, many studies since about 2005 have focussed on the potential adverse effects of sounds on fishes. A driving force in this change has been the need by regulators, industry, environmental groups and scientists to develop guidelines and criteria that can be used to assess whether particular sounds have deleterious effects on individual fishes or affect populations. There has also been a need to employ such information in regulations intended to protect fishes and ecosystems.

## 2 OVERVIEW

The purpose of this paper is to improve understanding of the issues related to the potential effects of anthropogenic sounds on fishes and to point to the need to examine effects not only on individual animals, but also to those on fish populations and ecosystems. However, since many readers may not be familiar with fish bioacoustics, we also include some background material to assist readers in understanding and interpreting data on the effects of anthropogenic sound. Accordingly, the paper starts with a brief discussion of underwater sound in order to introduce major concepts that are critical for understanding potential effects of anthropogenic sound. This is followed by a discussion of fish bioacoustics for those not familiar with the topic. We then focus on data on the potential effects of anthropogenic sound on fishes. It will become clear that there are major gaps in our knowledge that need to be filled in order to facilitate the development of appropriate and effective sound exposure criteria and the guidelines to implement them. Indeed, it is critical to understand that current criteria are still provisional and that substantially more data are required before firm criteria can be set. The review therefore ends with suggestions as to the most critical current data needs.

This paper is not intended to be a complete review of all the literature. Rather, our focus is on the major issues related to potential effects of anthropogenic sound on fishes and to help readers understand those aspects that are especially important. However, we do provide further citations so that those interested can delve deeper into the growing literature on the topic and we include a number of recent reviews that refer to the wider literature. Furthermore, the papers we do include are those we think are amongst the most informative and critical to understanding the main issues. At the same time, we do include a number of papers that we see as being problematic, so that as well as providing insight into work that is critical to understanding the effects of sound on fishes, we also provide information on work that may lead to misunderstanding.

## 3 ADDITIONAL BACKGROUND INFORMATION

For those interested in broadening their understanding of general issues of fish bioacoustics (hearing, sound production, behaviour *etc*.), there are papers in a volume by Webb *et al.* (2008) as well as several more recent reviews (Ladich, 2014; Ladich & Fay, 2013; Mickle & Higgs, 2018; Putland *et al.,* 2018). More detailed reviews of potential effects of anthropogenic sound on fishes (and other aquatic animals) can be found in papers by the authors of this review (Hawkins *et al.,* 2015; Hawkins & Popper, 2014; Popper & Hawkins, 2018) and in the reports of several meetings on the *Effects of Noise on Aquatic Life* (http://www.an-2019.org; Hawkins *et al.,* 2008; Popper & Hawkins, 2012, 2016 and the open access *Proceedings of Meetings on Acoustics* (http://www.go.umd.edu/UcA). Finally, a general overview of effects of anthropogenic sound on animals is provided by Slabbekoorn *et al.* (2018).

## 4 THE IMPORTANCE OF HEARING

Of all the senses, hearing provides fishes with information, often from great distances, in the widest variety of environments, by day and night and from all directions around the animal. The limitations of other senses such as vision, touch, taste and smell in the aquatic environment, particularly in providing rapid, long‐distance and 3‐D information, make sound an exceptionally important cue for many (perhaps most) aquatic animals.

Detection of the acoustic scene (often referred to as the soundscape), which is the ensemble of ambient sound, including sound events, associated with a specific location at a particular time, is found in all vertebrates (Bregman, 1994; Fay, 2009; Slabbekoorn, 2018). Indeed, many of the most important aspects of hearing are likely to have evolved to enhance analysis of the soundscape. For example, the ability to determine the direction of a sound (sound source localisation) enables fishes (and other vertebrates) to locate predators and move away from them or detect potential prey and move towards them (Hawkins & Popper, 2018; Sand & Bleckmann, 2008). Likewise, the ability to discriminate between different sounds enables fishes to tell friend from foe or recognise and select members of their own species for mating. Once hearing evolved in fishes, acoustic communication became possible. However, while sound production is found in some fishes, many, including some that hear very well (*e.g*., many otophysans), do not produce sounds. Instead, these species use hearing primarily for detection of those natural sounds that make up the acoustic scene. Because of the importance of sound to fishes, it becomes clear that any interference with detecting the acoustic scene or with those sounds used by some fishes to communicate, has the potential to affect fitness and survival!

## 5 UNDERWATER SOUND

While the basic physics of sound in water are similar to those in air, the density of the medium is greater and as a result sound travels about 4.8 times faster than in air (1500 m s^−1^ *v*. 343 m s^−1^). As a result, a 100 Hz sound has a wavelength of 3.43 m in air, but it is 15 m in water (see http://www.dosits.org for an excellent primer on underwater sound). While we do not go into underwater acoustics in any detail, a number of terms and ideas are presented since they are critical to understanding fish bioacoustics and the analysis of sounds that have the potential to affect fishes.

### 5.1 Acoustic terminology

It is important to distinguish between sound and vibration. Sound is generated by the movement of an object, such as a loudspeaker, or a pile being driven, in a medium such as air or water (Urick, 1983). The term vibration refers to the actual motion of the sound source. As the sound propagates from the source it can be detected as the pressure fluctuations in the medium, above and below the local hydrostatic pressure (the sound pressure). However, sound is also accompanied by a back‐and‐forth motion of the medium, referred to as the particle motion. (For a clear visualisation of sound pressure and particle motion see: http://www.dosits.org/science/sound/what-is-sound/).

The term noise is often used to describe unwanted sounds that are considered to be unpleasant, loud or disruptive to hearing, or that can hinder detection of a particular signal. In some cases, however, the terms ambient noise or background noise may also be used, as it is in this paper, to describe sound generated by natural sources, as well as by anthropogenic sources, especially where they may interfere with the detection of animal and other sounds.

### 5.2 Sound pressure, particle motion and the substrate

Sound pressure is a scalar quantity that acts in all directions. It can be described in terms of its magnitude, as well as its temporal and frequency characteristics. In contrast, particle motion is a back‐and‐forth motion and, as such, is a vector quantity. Accordingly, particle motion is described not only by specifying its magnitude and temporal and frequency characteristics, but also its direction of motion.

Sound pressure is expressed in SI units of pascals (Pa) or micropascals (μPa). Particle motion may be expressed in terms of the particle displacement (SI unit: metre m), or its time derivatives: particle velocity (meter per second m s^−1^) or particle acceleration (meter per second squared (m/s^2^). Sound intensity is the product of the sound pressure and the particle velocity, for which the SI units are watts m^−2^.

A fundamental point is that all fishes (including elasmobranchs) detect and use particle motion, particularly at frequencies below several hundred Hz (Nedelec *et al.,* 2016; Popper & Hawkins, 2018). Thus, the detection of particle motion is integral to hearing in all fishes (and invertebrates) and it is used to locate the direction of the source, even in those fishes that are also sensitive to sound pressure (Hawkins *et al.,* 2015; Nedelec *et al.,* 2016). As a consequence, when investigating the effects of sounds upon fishes, it is important to describe the sounds in terms of particle motion (Popper & Hawkins, 2018), as well as sound pressure. This may be done by measuring the particle motion directly (Amorim *et al.,* 2018; Mickle *et al.,* 2018; Roberts & Breithaupt, 2016) or by conducting experiments under free‐field acoustic conditions, where the particle motion can be predicted from measurements of the sound pressure (Hawkins *et al.,* 2014). Until recently, most studies of sound and fishes have only included measurement of the sound pressure and very few have considered particle motion in a biologically relevant context. This was not just because investigators did not fully appreciate the importance of particle motion, but also because of the difficulty in obtaining instrumentation to measure the particle motion (*e.g*., Lumsdon *et al.,* 2018; Martin *et al.,* 2016).

While it is possible to estimate particle velocity from measurements of the sound pressure (or by measuring the pressure gradient), this can only be done in locales that are distant from reflecting boundaries (the water surface or bottom) or other acoustic discontinuities (MacGillivray *et al.,* 2004), since such surfaces have significant influence on the sound field and thus, on the levels and directionality of particle motion. Under such conditions, sensors are needed that not only detect particle motion (whether particle displacement or its time derivatives: particle velocity or particle acceleration) *per se* but are also able to detect the vector components in three dimensions.

Passage of sound and vibration into the substrate, which can be caused by sources such as pile driving, dredging and seismic surveys, may result in waves propagating through the substrate, both as compression waves and interface waves (Popper & Hawkins, 2018). The interface waves are often referred to as ground roll (Hazelwood *et al.,* 2018). These waves travel slower than the speed of sound and can have strong particle motion components. They may also generate evanescent sound pressure and particle motion waves that propagate through the water.

### 5.3 Sound metrics

It is very important to always refer to a sound using the proper measures, or metrics, that best describes that sound.

#### 5.3.1 Continuous sound

Continuous sound (*e.g*., from shipping) is generally presented as the root mean square (dB_rms_) sound pressure or particle motion level, measured over a specified time interval, for a specified frequency range. The roughness of continuous sounds may be especially important when considering effects, using a statistic often called kurtosis (Henderson & Hamernik, 2012). However, while of potential importance, and while mentioned more and more frequently, kurtosis has yet to be applied to fish (or marine mammal) bioacoustics.

#### 5.3.2 Impulsive sounds

Impulsive sounds (*e.g*., from pile driving) are best presented as the instantaneous peak level, the dB_peak_. That is, the level of the zero‐to‐peak sound pressure or particle motion. Alternatively, the total energy within the pulse may be described by the sound exposure level (SEL; Popper & Hastings, 2009). The SEL is the integral, over time, of the squared sound pressure, normalised to a reference time of 1 s. The SI unit of sound exposure is the Pascal squared for 1 s (Pa^2^ s^−1^). The SEL may be specified for a single impulse or strike (the SEL_ss_). However, when impulsive sounds are repeated, for example when fishes are exposed to pile driving for a long period, it is appropriate to estimate the cumulative SEL (SEL_cum_) associated with a series of pile strikes. The SEL_cum_ is the total noise energy to which the animal is exposed over a defined time period (Popper & Hastings, 2009).

Another important characteristic of impulsive sounds is the rise time, which is the time a signal takes to increase from 10% to 90% of its highest peak value. The rise time may affect the response of animals and may be especially important in terms of injury, where sharp rise times may be especially damaging.

#### 5.3.3 Frequency spectrum

The frequency spectrum is also important. The sound pulse is composed of a range of frequencies, expressed in terms of the level at each frequency measured over a given bandwidth. The bandwidths utilised are generally 1 Hz or ^1^/_3_ octave (an octave is a doubling of frequency). It is important to specify the frequency bandwidth as different animals respond to different frequency ranges.

## 6 NATURAL SOUNDS IN THE AQUATIC ENVIRONMENT

### 6.1 Ambient sound

Aquatic environments are rarely silent. Ambient sound (often termed ambient noise) consists of sounds generated by physical sources such as wind, waves, precipitation and ground movement (geophony), together with biotic sounds (biophony) produced by a variety of marine organisms, including mammals, fishes and invertebrates. Examining the soundscape involves describing the characterisation of ambient sound in terms of its spatial, temporal and frequency attributes and the types of sources contributing to the sound field.

### 6.2 Fish sounds

Of the more than 33,000 species of fish, at least 800, from over 100 families, are known to produce sounds (Bass & Ladich, 2008). It is likely that with more studies, including freshwater fishes, many additional species will be shown to be sound producers. Many commercially important fish species produce sounds, including the Atlantic cod, *Gadus morhua* L. 1758 and haddock, *Melanogrammus aeglefinus* (L. 1758), both Gadidae (Hawkins *et al.,* 1974; Hawkins & Chapman, 1966) and many croakers and drums (Sciaenidae; Ramcharitar *et al.,* 2006). Sounds are produced in a wide range of contexts such as feeding, mating, or fighting (Hawkins & Myrberg Jr, 1983; Moulton, 1963). The detection of sounds may be used by female fishes to locate vocal males and identify suitable mates (Casaretto *et al.,* 2015). As a consequence, anything, including sounds from anthropogenic sources, that impedes the detection of these sounds can have an adverse effect on such fishes.

## 7 ANTHROPOGENIC SOUND SOURCES

There are many sources of anthropogenic sound in the sea, lakes and rivers, with quite different acoustical characteristics (Hawkins *et al.,* 2015; Popper *et al.,* 2014). Many commercial human activities introduce sound, either intentionally for a specific purpose, such as seismic surveys, or unintentionally as a by‐product of activities such as shipping and offshore and even onshore construction work. Coastal areas and areas where a high degree of human activity takes place, may be quite noisy; including harbours and shipping lanes. However, some high‐intensity sources of underwater sound, such as pile drivers and seismic airguns, can be detected over distances of several thousand kilometres. Thus, effects upon fishes may occur well away from the source itself.

There are two main classes of anthropogenic sound. Some sounds are transient or impulsive, while others are continuous. Impulsive sounds are often of short duration (generally well less than 1 s) and may show large changes in amplitude over their time course. They can either be single or repetitive. Examples of such sounds are those produced by seismic airguns, pile driving and underwater explosions. (Various anthropogenic sounds can be heard at: http://www.go.umd.edu/Ucd.) Most often, such sounds are only present over the course of a particular project and then end.

Continuous sounds are produced by shipping (both commercial and pleasure boats), operational wind turbines, seabed drilling *etc*. and may continue for months or even years (*e.g*., in a harbour or wind farm). A few of these, described below, are perhaps the most ubiquitous sounds potentially affecting fishes over the widest geographic areas. Sonar systems, while used very widely, generally operate within frequency ranges that are not detectable by fishes (Halvorsen *et al.,* 2012d; Popper *et al.,* 2007).

### 7.1 Seismic airguns

Airguns are impulsive sources used for seismic exploration for sub‐sea gas and oil reserves as well as for geological research (Gisiner, 2016). These devices use compressed air to produce a gas bubble which expands rapidly when released, creating a high intensity impulsive sound, primarily composed of energy below 200 Hz, but with the bulk of the sound from 20 to 50 Hz (Mattsson *et al.,* 2012). The sounds are directed downward into the seabed, though there is also some spreading laterally and they are reflected from various geological formations and then detected by a long array of hydrophones towed by the seismic vessel (see Gisiner, 2016 for a detailed description of seismic surveys).

### 7.2 Impact pile driving

Impact pile driving is widely used for the construction of bridges, harbours, wind farms and other offshore structures (Dahl *et al.,* 2015; Popper & Hastings, 2009). Striking by the hammer results in vibration of the pile in water and in the substrate, thereby generating sounds that potentially affect nearby animals (Dahl *et al.,* 2015; Hazelwood & Macey, 2016). The sounds produced by pile driving are impulsive, short (of the order of μs) and most of their energy lies below 500 Hz, though some energy may extend up to 1 kHz (Dahl *et al.,* 2015). The sound levels (both sound pressure and particle motion) vary substantially, depending on numerous factors such as pile diameter, hammer size, substrate characteristics, *etc*. The sounds produced by pile drivers are often very intense with SEL_ss_ often well‐exceeding 180 to 200 dB re 1 μPa^2^ s^−1^ and with very sharp rise times.

### 7.3 Other industrial activities

Many other industrial activities contribute to underwater noise. Such activities generally produce sound that has the most energy at low frequencies (*i.e*., <1 kHz). Dredging, for example produces high levels of broadband noise (de Jong *et al.,* 2016; Wenger *et al.,* 2017) and is used to extract sand and gravel from the seabed and from lakes, maintain shipping lanes and to install pipelines and cables within the seabed. Activities onshore, including the passage of vehicles, may increase noise levels in the sea, lakes and rivers, especially if they generate substrate vibration.

### 7.4 Operating wind turbines

Since *c*. 2000 there has been an enormous increase in the generation of electricity by wind farms located in coastal waters, especially in European seas. There is some concern that sounds from operating offshore wind turbines might affect fish behaviour, although the sounds generated are very different to those generated during wind‐farm construction (Cheesman, 2016). Most sound from a wind turbine is concentrated in a narrow band, centred around 180 Hz and the sounds are generally below about 700 Hz (Madsen *et al.,* 2006; Pangerc *et al.,* 2016). However, there is also a particle motion component to the sounds generated by wind turbines, accompanying substrate transmission (Sigray & Andersson, 2012; P. Gopu and J. Miller, personal communication, 2018), although this has rarely been monitored and has often been ignored. There is currently limited information available on the acoustic characteristics of offshore turbines, including those utilising tidal and wave energy (Lossent *et al.,* 2018; Schramm *et al.,* 2017).

### 7.5 Vessel noise

A significant proportion of anthropogenic noise in the ocean and other water bodies is created by motorised vessels, including large ships, fishing and pleasure boats (Pine *et al.,* 2016; Rossi *et al.,* 2016). Most vessels, and especially large ships, produce predominately low frequency sound (*i.e*., <1 kHz) from onboard machinery and hydrodynamic flow around the hull. Cavitation at propeller blade tips is also a significant source of noise across all frequencies (Ross, 1987, 1993). Low frequency sounds from ships can travel hundreds of kilometres and can increase ambient noise levels over large areas of the ocean (Ellison *et al.,* 2012; Southall, 2005).

Ambient noise levels in busy shipping lanes have recently increased (Hildebrand, 2009), across much of the frequency spectrum (Sertlek *et al.,* 2016), but especially at lower frequencies (<500 Hz; Erbe *et al.,* 2012; Bittencourt *et al.,* 2014). Large numbers of smaller pleasure and recreational vessels, including things like jet skis (Erbe, 2013), may also result in substantial increases in noise levels in coastal waters, lakes and rivers. Ice‐breaking ships can be a significant source of sound in polar regions.

## 8 FISH HEARING

### 8.1 Hearing capabilities

#### 8.1.1 Hearing sensitivity

There is a long history of fish hearing studies (Moulton, 1963; Tavolga, 1971). It is likely that all fishes (including elasmobranchs) detect sound and use it to learn about their environment (e.g., Ladich & Fay, 2013). Until recently, however, most studies have focussed on determination of hearing capabilities of fishes to sound pressure signals, despite it being clear that most fishes (and all elasmobranchs) primarily detect particle motion (Popper & Hawkins, 2018). (As an aside, lampreys (Petromyzontidae) also have an ear that has many characteristics in common with other vertebrates and both morphological (Popper & Hoxter, 1987) and recent physiological results (Mickle *et al.,* 2018) suggest that they only detect particle motion). There is a need to investigate the hearing abilities of lampreys and many other fishes, under conditions where the particle motion can be monitored or estimated and the ratios of these two potential stimuli can be varied. Such experiments have been reviewed in a number of recent papers, including Hawkins (2014) and Putland *et al.* (2018).

In addition to not focussing on particle motion, many studies have been conducted in tanks, or in poorly designed enclosures in open waters (*e.g*., the experiments by Debusschere *et al.*, 2016, which examined effects of pile driving during off‐shore wind‐farm construction on young European sea bass *Dicentrarchus labrax* (L. 1758) placed in glass 500 ml vials). In such environments, the sound fields presented to the fish are generally very complex and quite unlike the sound fields that a fish would encounter in a normal aquatic environment (Rogers *et al.,* 2016). As a result, such experiments often leave open questions regarding the actual nature of the sound field to which the animals were exposed and the stimuli to which they responded (Hawkins *et al.,* 2015). Ideally, hearing experiments should be carried out in specially designed tanks (Duncan *et al.,* 2016; Hawkins & MacLennan, 1976; Rogers *et al.,* 2016) or in natural aquatic environments, where both the particle motion and the sound pressure levels can be monitored precisely.

Keeping these caveats in mind, it is possible to get some appreciation of hearing capabilities of fishes. For example, every species studied to date is able to hear. In addition, the majority of fishes detect sounds from <50 Hz, even as low as 10–30 Hz, or even lower (Sand & Karlsen, 2000) to perhaps 300–500 Hz. Fishes that can detect sound pressure hear to perhaps 1000 Hz. And, a much smaller number of species have specialisations that enable them to detect sounds to 3–4000 Hz (Ladich & Fay, 2013).

**Figure 1:**
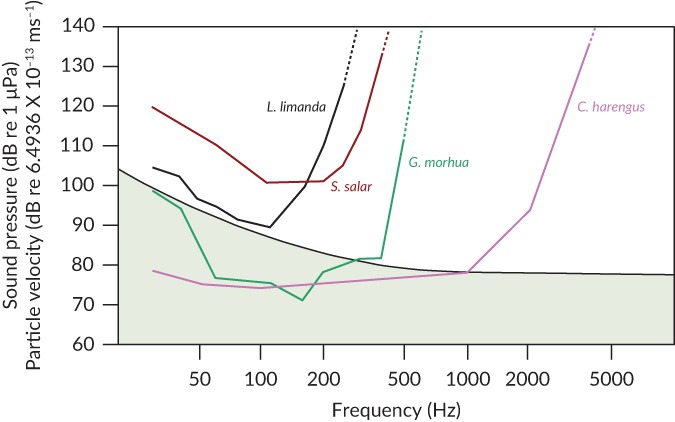
Fish hearing sensitivity (thresholds) obtained under open sea, free‐field, conditions in response to pure tone stimuli at different frequencies. The lower the thresholds (y‐axis), the more sensitive the fish is to a sound. Thus, *Clupea harengus* has best hearing of all of these species over a wider range of frequencies. Note that the thresholds in *Gadus morhua* and *C. harengus* obtained under quiet conditions may be below natural ambient noise levels, especially at their most sensitive frequencies. In the presence of higher levels of noise, the thresholds would be raised, a phenomenon referred to as masking. *Gadus morhua* and *C. harengus* are sensitive to both sound pressure and particle motion, whereas *Limanda limanda* and *Salmo salar* are only sensitive to particle motion. The reference level for the particle velocity is based on the level that exists in a free sound field for the given sound pressure level. *n.b*., For the particle velocity levels in this figure to match the sound pressure levels in a free sound field it is necessary to calculate an appropriate particle velocity reference level. If the standard reference levels are used, then the curves will not match one another and so they are not included here to keep the figure relatively simple. Fig. © 2018 Anthony D. Hawkins, all rights reserved

Because relatively few experiments on the hearing of fishes have been carried out under suitable acoustic conditions, valid data that provide actual hearing thresholds are available for only a few species (thresholds are generally defined as the lowest level of sound that can be detected 50% of the time). Figure 1 shows the measures of hearing, expressed as audiograms. determined in the open‐sea, rather than in a laboratory tank, for: the flatfish common dab *Limanda limanda* (L. 1758) (Chapman & Sand, 1974); the Atlantic salmon, *Salmo salar* L. 1758 (Hawkins & Johnstone, 1978); the *G. morhua*; (Chapman & Hawkins, 1973); the Atlantic herring, *Clupea harengus* L. 1758 (Enger, 1967). The *L. limanda* and *S. salar* are only sensitive to particle motion and have a relatively narrow bandwidth of hearing (up to *c*. 300–500 Hz), whereas species like *G. morhua*, where the gas‐filled swimbladder is close to the ear, are sensitive to sound pressure and show an increased hearing bandwidth (Fay & Popper, 1974; Sand & Hawkins, 1973).

It is important to understand that the swimbladder (and other gas‐filled cavities) potentially plays a major role in fish hearing. This is because the gas within the swimbladder is compressible and changes volume in response to fluctuating sound pressures (sound) and this results in the swim bladder serving as an acoustic transformer, translating sound pressure into re‐radiated particle motion (Sand & Hawkins, 1973). This produces higher levels of particle motion at the ears that stimulates the otolith organs (Popper *et al.,* 2003). Thus, having a gas bubble or a swimbladder close to, or connected to, the ear enhances the hearing abilities of fishes since the ear is not only stimulated directly by the particle motion component of the sound, but also indirectly by the particle motion reradiated from the gas bubble to the ear in response to sound pressure. The actual contribution of the indirect stimulation varies by species and depends on the distance between the bubble and the ear. For example, in *G. morhua*, hearing at low frequencies (<110 Hz), is based on the detection of particle motion, but at higher frequencies it is based on sound pressure due to the closeness of the anterior end of the swimbladder to the ear. Indeed, deflation of the swimbladder in *G. morhua* reduces sensitivity to sound pressure (Sand & Enger, 1973) and similar results have been shown for the goldfish *Carassius auratus* (L. 1758) (Fay & Popper, 1974).

In contrast, species like *S. salar*, despite having a swim bladder, are only sensitive to particle motion since the swimbladder is more distant from the ear (Hawkins & Johnstone, 1978; Knudsen *et al.,* 1992). Other species, such as *C. harengus* (as all Clupeiformes) has a specialised connection between a gas bubble as the ear and shows sensitivity to a much wider range of frequencies and this can extend to >100 kHz in clupeids of the shad family Alosinae (Mann *et al.,* 1998; Mann *et al.,* 2001). Finally, species that do not have a swimbladder or other gas bubble, such as flatfishes, some scombrids and some gobies, only detect particle motion and hear over a narrower bandwidth than *G. morhua*.

In addition to having a gas bubble that improves hearing sensitivity and bandwidth, a number of fish species have additional adaptations that mechanically link the swimbladder to the ear, thereby carrying the motion of the swimbladder to the ear without attenuation of the signal as a result of distance of travel. Best known of these adaptations are the Weberian ossicles, a series of bones that connect the swimbladder to the inner ear in otophysan fishes. (Popper *et al.,* 2003; Popper & Fay, 2011). In other species, the swimbladder has extensions that come close to, or may actually contact, portions of the inner ear and most notably to the saccule, the otolith organ most frequently associated with hearing (Ramcharitar *et al.,* 2006; Schulz‐Mirbach *et al.,* 2013).

#### 8.1.2 Limits to hearing sensitivity: masking

**Figure 2:**
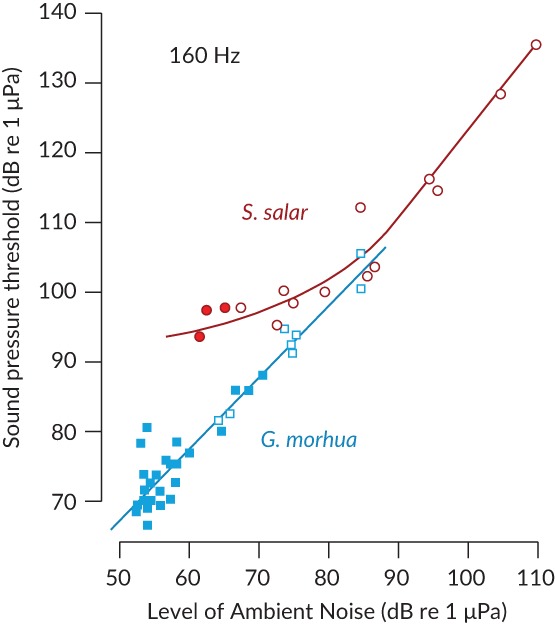
Masking in the *Gadus morhua* and *Salmo salar* by ambient noise. The thresholds were determined using a pure tone signal at a frequency of 160 Hz. The ambient noise (natural sea noise, augmented by white noise from a loudspeaker) is expressed as the spectrum level at that same frequency (dB re 1 μPa/Hz). Closed symbols, thresholds to natural levels of ambient noise; open symbols, thresholds to anthropogenic noise. *n.b*., The thresholds in *S. salar* were only influenced by high noise levels, above the natural ambient levels of noise (data from Hawkins, 1993). Fig. © 2018 Anthony D. Hawkins, all rights reserved

For the more sensitive fishes, hearing is not limited by the lowest level they can hear in a quiet environment, but by their ability to detect and discriminate biologically important sounds against the ambient noise background (Figures 1 and 2). In such conditions, the level of noise limits the lowest sound level that an animal can detect. This interference with detection of a biologically relevant sound by another sound, or noise, is generally known as masking and it is commonly found in all vertebrates, including fishes (Fay & Megela Simmons, 1999). As an example of masking, *G.morhua* only show best hearing sensitivity under the quietest sea conditions (Figure 2; Chapman & Hawkins, 1973). Any increase in the level of ambient sea noise results in a raising of the auditory threshold and a decline in the ability of the fish to detect, locate and recognise particular sounds. Critically, the masking of biologically relevant sounds occurs not only as a result of increases in natural ambient sea noise (caused by wind and rain) but also by any additional sounds added to the environment by humans. However, fishes that do not hear well may be less likely to have their hearing sensitivity affected by masking noise, since the lowest sound level they can detect may be above the level of the background noise (Hawkins & Johnstone, 1978).

Although the detection of sounds may be affected by the presence of masking sounds, it is also clear that fishes can use frequency filters to improve sound detection. They can also discriminate between different sound frequencies and intensities. They are also able to determine the direction from which sounds come (sound source localisation), a critical ability since this enables fishes to move towards potential food sources or away from predators (Fay, 2005; Fay & Megela Simmons, 1999; Hawkins & Popper, 2018; Sand & Bleckmann, 2008).

### 8.2 The ear

**Figure 3:**
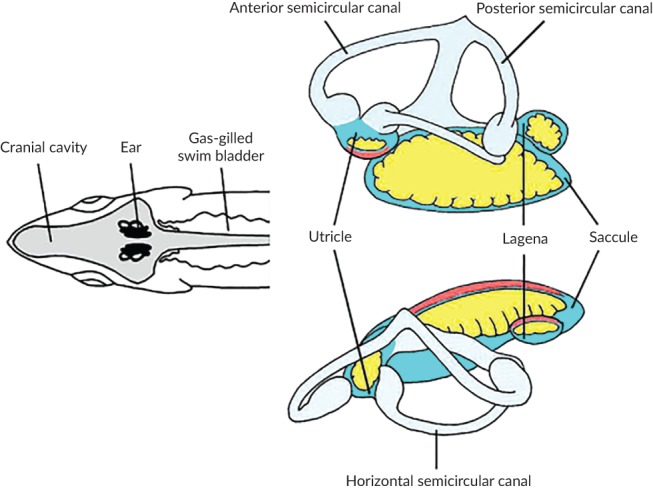
Schematic drawing of the ear of *Gadus morhua* (anterior is to the left): (a) top view of the body showing the location of the ears in the cranial cavity as well as the proximity of the rostral end of the swim bladder to the ear; (b) lateral and (c) top view of the same ear. Each ear is set at an angle relative to the midline of the fish. 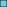, The otolith organs, 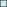, the semicircular canals (enlarged areas are the ampullae regions that contain the sensory cells); 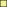, the dense calcarious otolith lying in close proximity to the sensory epithelium (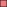). Also see Figure 4. Fig. © 2018 Anthony D. Hawkins, all rights reserved

**Figure 4:**
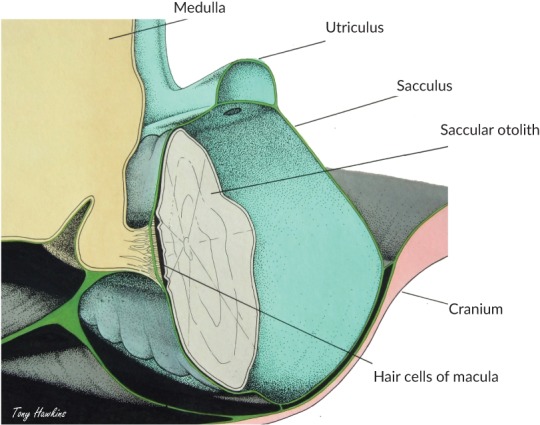
A frontal view of the head of *Gadus morhua* showing a section of the saccule (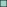). The saccular chamber is filled with perilymph and contains the otolith (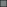), which lies close to the sensory hair cells of the epithelium (macula). The hair cells are innervated by the eighth cranial nerve. Fig. © 2018 Anthony D. Hawkins, all rights reserved

Fishes detect sound with paired inner ears (Figure 3), located in the cranial cavity lateral to the brain at the level of the medulla (Figures 3 and 4), that closely resembles ears found in other vertebrates. Since a fish's body is the same density as water, there is no need for any external structures (external or middle ears) to carry sound to the sensory regions of the ear. The ear consists of three semi‐circular canals and associated sensory regions (ampullae) that are primarily involved in detection of angular acceleration and three otolith organs (saccule, lagena, utricle) that are involved in hearing and positional senses (Popper *et al.,* 2003). There is very substantial variation in the morphology of the ears of fishes and particularly in the regions associated with hearing (Ladich & Schulz‐Mirbach, 2016; Retzius, 1881; Schulz‐Mirbach *et al.,* 2018; Schulz‐Mirbach & Ladich, 2016), leading to the suggestion that there is very substantial diversity in hearing mechanisms (and potentially capabilities) in different species (Popper *et al.,* 2003).

**Figure 5:**
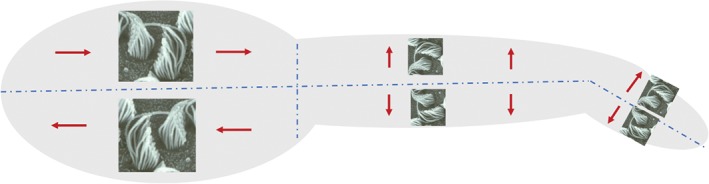
The sensory epithelia of the end organs of the inner ear have numerous mechanoreceptive sensory hair cells. The apical ends of these cells, directed into the lumen of the epithelia, have ciliary bundles (inserts in the figure) consisting of a single kinocilium (longest of the cilia) and graded stereocilia. Bending of the ciliary bundle during sound stimulation results in neurotransmitter release to stimulate the 8th cranial nerve. The sensory cells on the otolith maculae are organized into orientation groups, with all of the cells in each group having their kinocilia in the same general direction. In this typical saccular epithelium (anterior to the left, dorsal to the top), the cilia on the rostral end are oriented rostrally or caudally, while the cells on the caudal end are oriented dorsally and ventrally. 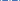, The approximate dividing lines between orientation groups)

The auditory parts of the ear, the otolith organs, each have a sensory epithelium that lies in close contact with a dense calcium carbonate structure, the otolith (Figures 3 and 4). The sensory epithelium (often referred to as a macula) has many sensory hair cells that are very similar to those found in the mammalian ear (Figure 5). When a fish is exposed to particle motion, the body, along with the sensory cells, move with the water, while the far denser otoliths move at a different amplitude and phase. This results in bending of the cilia on the apical surface of the sensory cells, releasing a neurotransmitter and sending a signal to the brain through an afferent neuron.

A critical role of the ear in fishes is involvement with determination of sound source direction (Hawkins & Popper, 2018). The sensory hair cells are morphologically polarised and the response of an individual cell changes with bending in different directions. Thus, each cell is directionally sensitive. Furthermore, the cells are organised into orientation groups in which all of the kinocilia are in the same direction (Figure 5). These hair cell orientation patterns, which vary in different species (Popper & Coombs, 1982), show graded responses to particle motion from various directions, thereby enabling a fish to determine direction by comparing information from different receptor groups (Fay, 2005; Hawkins & Popper, 2018; Sand & Bleckmann, 2008).

## 9 EFFECTS OF ANTHROPOGENIC SOUND

There are very few experimental examples of sound being sufficiently loud to result in death or mortal injury to fishes. However, far more importantly from the perspective of potential effects, is that anthropogenic sound, even at levels far lower than those that might result in mortality, may result in temporary hearing impairment, physiological changes, changes in behaviour and the masking of biologically important sounds (Table 1; Popper *et al.,* 2014; Erbe *et al.,* 2016). There may be significant consequences to individuals and populations as a result of changes in behaviour, including impairment of spawning, interference with foraging and feeding, or disruption of migrations and habitat selection. Exposure to sound may also (but not always) result in physiological changes that may include stress effects (Filiciotto *et al.,* 2016). However, as pointed out by Hawkins *et al.* (2015), there are large gaps in our knowledge of effects of sound on fishes that need to be filled if we are to fully understand the implications of exposure to anthropogenic sounds.

**Table 1:** Potential effects of anthropogenic sound on animals

Effect	Description
Death	Sound exposure results in instantaneous or delayed mortality.
Physical injury & physiological changes	Physical injury results in temporary or permanent impairment of the structure and functioning of some parts of the body. Physiological changes result in increased stress or other effects that can lead to reduced fitness.
Hearing threshold shift	Loss of hearing, temporarily or permanently, results in decreased ability to respond to biologically relevant sounds.
Masking	Noise results in a decrease in detectability of biologically relevant sounds (*e.g*., sounds of predators and prey, sounds of conspecifics, acoustic cues used for orientation).
Behavioural responses	Behavioural responses include any change in behaviour from small and short‐duration movements to changes in migration routes and leaving a feeding or breeding site. Such responses are likely to vary from species to species, depending on numerous factors such as the animals normal behavioural repertoire, motivational state, time of day or year, age of the animal, *etc*. Some changes in behaviour, such as startle reactions, may only be transient and have little consequence for the animal or population.
No obvious behavioural responses	Animals may show transient or no responses, even if they detect the sound (*e.g*., to a very low‐level sound) or habituation may take place. However, even if there is no response, there is always the possibility that physical injury and physiological changes may take place without the animal showing overt changes in behaviour

### 9.1 Effects upon behaviour

#### 9.1.1 Caveats in interpretation of laboratory studies

In evaluating data on potential behavioural effects of anthropogenic sound on fishes it is important to first appreciate a number of caveats that are critical to interpretation of various studies. In particular, one must be cautious when evaluating the results from behavioural studies done in tanks and even in larger enclosures (Hawkins & Popper, 2016b; Popper & Hawkins, 2018). The fundamental issue is that captive animals, no matter whether on land or in the water, often do not show the full range of behaviours observed in wild animals (Benhaïm *et al.,* 2012; Oldfield, 2011), especially if they have been bred in captivity (El Balaa & Blouin‐Demers, 2011; Petersson *et al.,* 2015). As a result, data from studies using free‐living fishes are very likely to differ from those with captive fishes because of the many subtle factors that determine their behaviour in a natural setting. Put another way, one must take very considerable caution in extrapolating behaviour from studies of captive animals to how wild animals may respond to the same stimulus.

A second critical caveat is that when studies are done in tanks and other enclosures the sound fields may be very different from those that fishes experience in the wild, especially in terms of the magnitude of particle motion relative to sound pressure (Duncan *et al.,* 2016; Gray *et al.,* 2016; Rogers *et al.,* 2016). Many fishes live close the substrate, or occupy burrows, coral reefs, mangroves and kelp forests, where sound transmission may be especially complex; while others may occupy open waters. None of these acoustic environments, however, are anything like that in a fish tank where walls are thin and often made of glass or flexible material. Consequently, the walls of tanks vibrate and set up highly perturbed sound fields that would have ratios of pressure and particle motion unlike those that an animal would ever encounter in the wild (Parvulescu, 1964; Rogers *et al.,* 2016). Thus, even if a fish shows a particular behaviour pattern in response to a tank‐based sound, the same sound produced in the wild may have very different acoustic characteristics and thus may or may not elicit the same behaviour as in the tank. It is important to monitor the particle motion as well as the sound pressure and where possible to ensure that the acoustic conditions under which experiments are conducted are similar to those the fish would experience in the wild. Where the particle motion is properly monitored as well as the sound pressure, some physiological experiments on captive animals may provide some useful information on the levels that produce particular effects. However, it is necessary to be circumspect in extrapolating the findings to wild animals.

Finally, in considering behaviour, it is also important to recognise that the responses of fishes may vary with their age and condition, as well as under different environmental conditions. Moreover, responses may vary with different sound sources, or with the same sound when the level of sound received by the animal differs (De Robertis & Handegard, 2013; Lucke *et al.,* 2016).

#### 9.1.2 Behavioural responses to sound

Sounds can have several different types of behavioural effects. Some fishes may react negatively to a sound. There may be changes in feeding or mating; migratory paths may be altered; and the finding of places for larval settlement may be disrupted. Anthropogenic sounds may interfere with detection of the overall acoustic scene (or soundscape) as well as affecting sound communication by fishes. Masking may result in lessened ability to detect biologically significant sounds and may also result in the generation of signals that are similar to those produced by the fish themselves (Kaplan *et al.,* 2015; Kaplan *et al.,* 2016; Pine *et al.,* 2016).

There has been a variety of studies of the potential effects of anthropogenic sound on fish behaviour. However, many of those studies must be considered with great caution since they were done in laboratory tanks, or on species, such as the zebrafish *Danio rerio* (Hamilton 1822), that appear to thrive in captivity, unlike many other species, and which are behaviourally and physiologically very different to the important commercial species such as salmonids, gadids, sciaenids, *etc*. Extrapolation from laboratory fishes to commercially important species must be done with the greatest caution.

At the same time, some observations from recent behavioural studies do provide instructive insight and guidance. For example, while it is generally assumed that fishes with better hearing abilities, are more likely to show behavioural responses to sounds than less sensitive species, this may not always be the case. Comparisons of laboratory responses of *D. rerio* and Lake Victoria cichlids, such as *Haplochromis piceatus* Greenwood & Gee 1969, to sounds, the former having better hearing sensitivity (lower auditory thresholds) and a wider frequency range than the latter, showed that both species exhibited a significant reduction in swimming speed in the first minute of exposure that were not obviously related to differences in their hearing abilities (Shafiei Sabet *et al.,* 2016). Similarly, Hawkins *et al.* (2014) showed that changes in the behaviour of schools of wild sprat *Sprattus sprattus* (L. 1758) and mackerel *Scomber scombrus* L. 1758, to sound playback took place at similar sound levels, despite major differences in their hearing abilities.

#### 9.1.3 Responses to continuous sounds

Many anthropogenic sources produce long‐duration signals that can increase the overall sound level in the environment for extended periods of time. Increased shipping in a harbour, increased pleasure boats on a reef, or continuous operation of an offshore wind turbine or oil rig, may change the acoustic environment to which a fish is adapted. Consequently, critical aspects of fish behaviour could be interfered with by the presence of long‐term sounds that mask a fish's ability to detect sounds of biological importance to the animals. A wide range of behaviour patterns may be affected by increased background noise. For example, anthropogenic sounds may interfere with foraging behaviour either by masking the relevant sounds or by resembling the sounds that the prey may generate (Purser & Radford, 2011). Similarly, fishes may avoid predators by listening for the sounds that the predators produce, either deliberately or inadvertently. Studies have shown that elevated sound levels, including intermittent or pulsed sounds, may affect predator prey interactions (Luczkovich & Keusenkothen, 2008; Remage‐Healey & Bass, 2006). It is evident that anthropogenic noise can affect predator avoidance in some fishes. At the same time, however, it must be kept in mind that all studies on predator avoidance to date have involved captive fish in enclosed environments. Clearly, there is a need to examine the behaviour of wild fishes under more natural conditions.

Another issue is that many fishes migrate to feeding areas or spawning grounds and may subsequently return to other locations. During migrations, fishes may use a variety of cues to orientate and navigate, including natural soundscapes. High level sounds may result in avoidance responses, deflecting fish away from their migration routes. For example, Montgomery *et al.* (2006) suggested that the ability of larval reef fishes to locate their home reefs by responding to their characteristic sounds might be affected by changes in the noise level (Stanley *et al.,* 2012). There are significant differences in the spectral and temporal composition of the ambient sound associated with different coastal habitat types (Radford *et al.,* 2010) and Gordon *et al.* (2018) recently pointed out that changes in habitats may negatively affect the auditory settlement behaviour of coral‐reef fishes. Acoustic cues guide the orientation, habitat selection and settlement of many fishes, but these processes may be impaired if degradation alters reef soundscapes.

Sounds are also important for many fish species for spawning. In particular, any interference with detection of spawning sounds can have a significant effect on reproductive success of a population. For example, Casaretto *et al.* (2015) showed that male *M. aeglefinus* are territorial and that visits to their territories by females, induced by the sounds of males, triggered courtship behaviour, leading to the spawning embrace It has been suggested by de Jong *et al.* (2017) that acoustic communication may play a crucial role in reproductive interactions and they point out that over 800 species of fish have been found to communicate acoustically.

In addition to affecting the detection of biologically important sounds, there is also limited evidence that anthropogenic sounds will result in fishes altering their own sounds to avoid masking (Radford *et al.* (2014). Similarly, Holt and Johnstone (Holt & Johnston, 2014; Holt & Johnston, 2015) investigated effects of elevated noise levels on a sound‐producing freshwater fish, the black‐tail shiner *Cyprinella venusta* Girard 1856, in tanks. When elevated levels of natural river noise were played back to the fish, it was found that several acoustic features of the fish calls were altered under noisy conditions. Most notable the spectral composition of the calls was altered by the fish (termed the Lombard effect).

#### 9.1.4 Observed effects from impulsive sound sources

Especially important are the sounds produced by impulsive sources. Such sounds are typically transient, brief (< 1 s), broadband and show high peak sound pressure with a rapid rise time and rapid decay. The greater amount of (still very limited) data available on behavioural responses to impulsive sound comes from studies of pile driving sounds. Moreover, most of these behavioural studies have been conducted on captive fish, maintained in confined spaces (Herbert‐Read *et al.,* 2017; Spiga *et al.,* 2017), though a few recent studies have been conducted on fishes in the wild (Hawkins *et al.,* 2014; Iafrate *et al.,* 2016; Roberts *et al.,* 2016a). For example, Hawkins *et al.* (2014) observed the behaviour of schools of *S. sprattus* and *S. scombrus* in mid water at a quiet coastal location, using an echosounder. *Sprattus sprattus* is sensitive to sound pressure, while the *S. scombrus* is likely to be sensitive only to particle motion. The fish were exposed to short sequences of repeated impulsive sounds, simulating the strikes from a pile driver, at different sound levels. Results showed that the incidence of behavioural responses increased with increasing sound level. The response levels suggested that both species would show changes in their behaviour at considerable distances (many kilometres) from a pile driving operation. However, the responses of *S. sprattus* at night were very different to those shown during the day. *Sprattus sprattus* schools break up at night and the individual fish did not respond to the playback of pile driving sounds at that time.Despite major differences in their hearing abilities the *S. sprattus* and *S. scombrus* responded in the daytime playback experiments to impulsive sounds at similar sound levels. This may be the result of *S. scombrus* being readier to respond to any stimulus, observations suggested that they were perhaps flightier than *S. sprattus*. However, this, like most other aspects of how fishes respond behaviorally to anthropogenic sound, still needs extensive study.

There have also been a number of studies of the response of captive demersal species to pile driving sounds. For example, Neo *et al.* (2014) found that that intermittent sounds may yield longer‐lasting behavioural effects than continuous sounds (Neo *et al.* 2015). Moreover, ramp‐up procedures, where sounds are slowly increased in level so as to warn fishes of impending sounds, do not necessarily lead to mitigation (Neo *et al.* 2016). At the same time, these studies were done in enclosures that did not resemble natural acoustic environments and many were done with *D. rerio*, a species that is small, thrives in small tanks and which hears far better than most (if not all) species likely to be exposed to pile driving operations.

Kastelein *et al.* (2015, 2017) determined acoustic dose–response relationships for behavioural responses to the play back of pile driving sounds by *D. labrax* in a netting enclosure within a very shallow rectangular pool, where the sound field was nothing like that in the wild. It was concluded that if wild *D. labrax* were exposed to pile driving sounds at the levels used in the study, there were unlikely to be any adverse effects on their ecology, because their initial responses were short‐lived. However, the experiments were carried out on fish that had spent their whole lives in captivity.

In a more detailed series of experiments on laboratory‐bred juvenile *D. labrax*, Radford *et al.* (2016) exposed fish to playbacks of pile driving sounds and seismic sounds in laboratory‐based studies intended to examine how an initial response to different sound types potentially changes over time. The study found a lessened response after repeated exposure to pile driving sound and it was concluded that this was probably due to increased tolerance (habituation), or a shift in hearing threshold (temporary threshold shift; TTS or permanent threshold shift; PTS) following initial exposure.

Roberts *et al.* (2016a, 2016b) examined the responses of a number of wild demersal species to the playback of pile driving sounds and elicited behavioural responses including startle responses and directional avoidance. The exposure levels were similar to the 50% response levels determined by Hawkins *et al.* (2014) for schools of *S. sprattus* and *S. scombrus* using the same sound projector array. However, Roberts *et al.* (2016b) emphasised that while the water‐borne component of the sound was accurately reproduced by the sound projectors, the projectors were not able to replicate the additional substrate‐borne vibrations that pile drivers produce.

The conclusion from all of these studies is that we really know very little as to how fish behave in the wild to impulsive signals. This is because most studies were done in the laboratory where the sound stimulus is of great question and where fishes cannot show natural behaviour. Moreover, there was considerable variation in species, age of fish and whether the animals were raised in captivity or not. Nevertheless, there have been studies that examined the behavioural responses of large groups of fishes to the impulsive sound of seismic surveys in the wild. However, these studies, unlike the ones cited earlier, were not designed to examine the behaviour of individual or small groups of fishes. Instead, these studies examined changes in the distribution of wild fishes in the presence of an actual seismic survey. The horizontal and vertical distributions of both pelagic and demersal fishes have been shown to change during and after airgun operations (Løkkeborg *et al.,* 2012), although they generally returned to the original site within hours or days after the end of the seismic operation (Engås *et al.,* 1996; Engås & Løkkeborg, 2002). Other studies have shown that fish may respond to approaching vessels by diving towards the seafloor or by moving horizontally out of the vessel's path, with reactions often initiated well before the vessel reaches the fish (Ona *et al.,* 2007).

### 9.2 Effects upon hearing sensitivity

Exposure to sounds may result in hearing loss as a result of damage to the sensory cells of the inner ear or the innervating neurons. While temporary hearing loss (TTS) occurs in fishes, there is no evidence for permanent hearing loss (PTS). Indeed, PTS may not occur in fishes since they can repair or replace sensory hair cells of the inner ear that have been lost or damaged (Smith *et al.,* 2006; Smith & Monroe, 2016). TTS is a short duration decrease in hearing sensitivity resulting from exposure to intense sounds or sounds of long duration. After termination of the sound, normal hearing ability returns over a period that may range from minutes to days, depending on many factors, including the intensity and duration of exposure (Amoser *et al.,* 2004; Smith *et al.,* 2006; Smith & Monroe, 2016). However, during a period of TTS, animals may be placed at some risk to survival in terms of poorer communication, inability to detect predators or prey and difficulty in assessing their environment.

TTS has been demonstrated in a number of fish species from a diverse array of sounds (Smith & Monroe, 2016) but in all cases, TTS was only found after multiple exposures to intense sounds (*e.g*., < 190 dB re 1 μPa rms) or as a result of long‐term exposure (*e.g*., tens of minutes or hours) to somewhat less intense sounds. Even when a signal source caused TTS in some individuals or species, it did not occur in other specimens or other species (Popper *et al.,* 2005; Popper *et al.,* 2007). In most cases, normal thresholds returned within a few hours to several days. There is also evidence that, given the same type and duration of sound exposure, a much more intense sound will be required to produce TTS in fishes that do not hear well compared with fishes that do hear well (Popper *et al.,* 2007; Smith *et al.,* 2004). Since TTS can arise from prolonged exposure to sound (though this is not always so), it is not likely to be of great significance for fishes that are only briefly exposed to a source (Halvorsen *et al.,* 2013; Popper *et al.,* 2007).

Of far greater concern is that TTS may occur when there is long‐term noise exposure such as in harbours and other areas where there is a long‐term increase in sound level. While limited, TTS is correlated with damage to sensory hair cells of the ear and it has been shown that recovery from TTS occurs in parallel with repair or replacement of sensory cells (Smith *et al.,* 2006; Smith *et al.,* 2011). Other studies have shown that exposure to intense sound may result in hair cell damage, but they did not examine whether this was accompanied by a loss of hearing (Casper *et al.,* 2013b; Enger, 1981; Hastings *et al.,* 1996; McCauley *et al.,* 2003). At the same time, studies of other species or other types of intense sounds have not resulted either in TTS or hair cell damage (*e.g*., Halvorsen *et al.,* 2013; Popper *et al.,* 2005; Popper *et al.,* 2007).

Clearly, there is still a question as to whether TTS occurs in fishes exposed to anthropogenic sounds and, if so, which sounds will result in TTS. Moreover, there appears to be broad species variation as to whether TTS will occur and there is even evidence that different genetic stocks of the same species may or may not show TTS (Halvorsen *et al.,* 2013; Popper *et al.,* 2007). Moreover, none of the studies on TTS to date have determined whether the loss of hearing (or lack of loss of hearing) is correlated with exposure to sound pressure or particle motion. Finally, none of the studies have been done on wild animals where there is the potential to escape from areas of intense sounds, or to test whether a small change in hearing threshold has any real impact on fitness (Popper *et al.,* 2014).

### 9.3 Stress

Animals showing no overt sign of responding to an environmental stimulus may, nonetheless, experience physiological changes that are often referred to as stress responses. These are often similar to stress effects to sound exposure found in terrestrial animals (Gourévitch *et al.,* 2014; Kight & Swaddle, 2011; Weilgart, 2017; Wysocki *et al.,* 2006). Stress may include hormonal, autonomic, immune and behavioural responses that may initially allow fishes (as other animals) to adapt to adverse conditions. However, some stressors may change the state of physiological processes and affect homeostasis, thus having an adverse effect upon the animals’ health and well‐being. Very little is known about stress effects in fishes and the significance of such effects in response to anthropogenic sounds is even less clear (Tennessen *et al.,* 2016).

There is an increasing body of literature on potential stress effects of exposure to both continuous and impulse anthropogenic sounds (Buscaino *et al.,* 2010; Celi *et al.,* 2016; Nedelec *et al.,* 2015; Sierra‐Flores *et al.,* 2015). However, as for behavioural studies, there is a wide range of species used, a diverse set of exposure paradigms, very different results depending on species and paradigm, and, most importantly, all of these studies have been done in the laboratory. Consequently, one must be cautious in extrapolating to how a fish might respond to a stressor in the wild where the fish's movement is not restrained and it could, potentially, move away from a stressor. It is also important to distinguish between normal or tolerable variations in response to environmental stress from those changes that will have consequences for survival and reproduction. At present, critical examination of these long‐term changes in fishes as a result of sound exposure is lacking.

In considering potential physiological effects, a critical issue is that potential effects of sounds on the physiology of fishes, as measured by various stress parameters, are quite variable and are not particularly instructive with regard to how exposure might affect fishes. In particular, all of the studies to date, including both long and short‐term exposures, were made on captive animals in enclosed areas where the fishes could not avoid the sounds. Thus, the acoustics were different than those an animal would encounter in the wild and the fish could not move away from the disturbing sound. Thus, it is possible that it is not the sound itself that resulted in the stress response, but the inability of the animals to move away from the sound.

### 9.4 Death and injury

Death and injury of fishes are probably the most easily observed responses to high levels of anthropogenic sound. However, there are only the most limited data on mortality in fish from sound exposure and these are when animals are very close to pile driving sources (California Department of Transportation, 2001), but not for other sound sources. Indeed, exposure of fishes to very high intensity low and mid‐frequency sonars resulted in no mortality (Halvorsen *et al.,* 2013; Popper *et al.,* 2007), nor did exposure to seismic airguns (Popper *et al.,* 2005; Popper *et al.,* 2016). There are, however, some data showing that fishes receiving high intensity and particularly impulsive, sounds will experience damage to body tissues. This damage appears to result from rapid oscillation of the walls of the swimbladder when stimulated by an impulsive source. In such cases, it appears that the swimbladder expands and contracts rapidly, thereby damaging the proximate organs including liver, kidney, gonads and the swimbladder itself (Halvorsen *et al.,* 2012b; Halvorsen *et al.,* 2012c). For example, of five species exposed to high intensity simulated pile driving signals (Casper *et al.,* 2013a; Halvorsen *et al.,* 2012b; Halvorsen *et al.,* 2012c), only the hogchoker *Trinectes maculatus* (Bloch & Schneider 1801), a flatfish without a swim bladder, showed no tissue damage (Halvorsen *et al.,* 2012b). At the same time, exposure to very high intensity continuous signals that did not contain any impulsive components showed no tissue damage in five different species (Halvorsen *et al.,* 2012d; Halvorsen *et al.,* 2013; Kane *et al.,* 2010; Popper *et al.,* 2007).

A recent set of studies, using a pile driving sound as a stimulus, enabled investigators to quantify the physical effects of sound exposure on various tissues (Casper *et al.,* 2012; Casper *et al.,* 2013a, 2013b; Casper *et al.,* 2017; Halvorsen *et al.,* 2012a, 2012b, 2012c; Popper *et al.,* 2013). While these results directly relate to pile driving, they are also likely to give guidance for potential effects of other impulsive sounds on fishes and so they have been incorporated into the most recent guidelines for fishes on interim sound exposure criteria (Table 2; Popper *et al.,* 2014; Andersson *et al.,* 2017).

**Table 2:** Proposed interim criteria for mortality and recoverable injury from exposure to pile driving signals are based on 960 sound events at 1.2 s intervals (Halvorsen et al., 2012b, 2012c). Temporary threshold shift (TTS) based on Popper et al. (2005). The same peak levels are used both for mortality and recoverable injury since the same sound exposure level (SEL_ss_) was used throughout the pile driving studies. All criteria are presented as sound pressure even for fishes without swim bladders since no data for particle motion exist. Relative risk (high, moderate, low) is given for animals at three distances from the source defined in relative terms: N, near; I, intermediate; F, far (from Popper et al., 2014)

Type of Animal	Mortality and potential mortal injury	Impairment			Behaviour
		Recoverable injury	TTS	Masking	
Fish: no swim bladder (particle motion detection)	> 219 dB SEL_cum_ or > 213 dB peak	> 216 dB SEL_cum_ or > 213 dB peak	>>186 dB SEL_cum_	(N) Moderate (I) Low (F) Low	(N) High (I) Moderate (F) Low
Fish: swim bladder is not involved in hearing (particle motion detection)	210 dB SEL_cum_ or > 207 dB peak	203 dB SEL_cum_ or > 207 dB peak	> 186 dB SEL_cum_	(N) Moderate (I) Low (F) Low	(N) High (I) Moderate (F) Low
Fish: swim bladder involved in hearing (primarily pressure detection)	207 dB SEL_cum_ or > 207 dB peak	203 dB SEL_cum_ or > 207 dB peak	186 dB SEL_cum_	(N) High (I) High (F) Moderate	(N) High (I) High (F) Moderate
Sea turtles	210 dB SEL_cum_ or > 207 dB peak	(N) High (I) Low (F) Low	(N) High (I) Low (F) Low	(N) High (I) Moderate (F) Low	(N) High (I) Moderate (F) Low
Eggs and larvae	> 210 dB SEL_cum_ or >207 dB peak	(N) Moderate (I) Low (F) Low	(N) Moderate (I) Low (F) Low	(N) Moderate (I) Low (F) Low	(N) Moderate (I) Low (F) Low

Peak and rms sound pressure levels dB re 1 μPa; SEL dB re 1 μPa^2^ s^−1^.

In brief, results from these studies showed a general correlation between the extent of tissue damage and the cumulative level of sound energy to which fish were exposed. For example, there was no tissue damage in one of the main study species, Chinook salmon *Oncorhynchus tshawytscha* (Walbaum 1792), following exposure to sounds below an SEL_cum_ of 210 dB re 1 μPa^2^ s^−1^. At an SEL_cum_ that was a few dB higher (but with sounds given over the same time period), internal injuries started to appear and when the level reached 219 dB re 1 μPa^2^ s^−1^ there were massive internal injuries that would likely result in death. Studies with other species showed that while there is some variation in SEL_cum_ required for onset of physiological effects, this is always at SEL_cum_ levels >203 dB re 1 μPa^2^ s^−1^ (Casper *et al.,* 2013a; Halvorsen *et al.,* 2012b).

At the same time, results show that the effects do not support the idea of an equal energy hypothesis, which is an idea based on the premise that the same effect will show up as long as the total energy to which a fish is exposed remains the same (Woodbury & Stadler, 2008). Instead, experimental results clearly show that the degree of effect depends upon a combination of the energy within single strikes (SEL_ss_) and the number of strikes, but the effect is not predictable from just knowing the cumulative energy (Casper *et al.,* 2016; Halvorsen *et al.,* 2012c).

Studies subsequently found that *O. tshawytscha* and hybrid white bass *Morone chrysops* (Rafinesque 1820) x striped bass *Morone saxatilis* (Walbaum 1792), recovered from all apparent physical effects within 10 days of exposure (Casper *et al.,* 2012, 2013a). However, it was made clear that recovery took place in the laboratory and that animals in the wild with similar injuries would have lower fitness and be more susceptible to predation and disease until they fully recovered. This is a concrete example of the need to be cautious in interpreting the results of laboratory experiments.

An additional question was whether hearing was affected by exposure to up to 960 sequential simulated pile strikes. Limited data showed that damage to ear tissues did not show up until the SEL_cum_ was 216 dB re 1 μPa^2^ s^−1^ (Casper *et al.,* 2013b). However, both species studied have swim bladders that terminate some distance from the ear and so movement of the swimbladder walls would not directly affect the inner ear. It is possible that fishes with gas‐filled organs near or directly associated with the ear would show damage at lower sound exposure levels due to the impulsive movement of the organ walls, much as they damage other nearby tissues.

## 10 EFFECTS ON FISH POPULATIONS AND THE WIDER ECOSYSTEM

The studies described previously have largely dealt with effects upon individual animals. However, for fishes, unlike marine mammals, perhaps the greater concern lies with effects upon populations rather than individuals (Hawkins & Popper, 2016a; Pirotta *et al.,* 2018). The extent to which sound affects the structure and functioning of fish populations and ecosystems, both marine and freshwater, is probably of considerable importance, although such effects have yet to be established.

Attempts to model changes in population parameters were first addressed for marine mammals. The population consequences of acoustic disturbance (PCAD) approach (NRC, 2005), recognises that there may be significant effects at individual, population and ecosystem levels. The population consequences of disturbance (PCoD) approach (Harwood *et al.,* 2014) is a formal, mathematical version of the PCAD model that uses the opinions of experts to quantify the transfer functions that describe the relationships between the different compartments of the PCAD model. It provides a protocol that can be used by regulators and developers to examine how sound exposure might impair the ability of individual animals to survive, breed, reproduce, or rear young and to quantify how this impairment may affect the abundance of the species concerned.

For species where there is limited knowledge of ecological interactions, an alternative risk assessment tool is required. Fisheries biologists have recently considered new risk‐based approaches in assessing the effects of fishing upon species for which there are only limited data on key population parameters. The productivity susceptibility assessment (PSA) approach (Patrick *et al.,* 2010) has been applied to fish stocks to determine the effect of human activities upon fishes. Such an approach attempts to evaluate the vulnerability of fish stocks to fishing; based on their biological productivity and potential for resisting adverse effects. This approach has been increasingly used to identify species at risk within multispecies fisheries (Hobday *et al.,* 2011; Smith *et al.,* 2007) and may have wider applicability in assessing risks from noise exposure.

## 11 SOUND EXPOSURE CRITERIA AND GUIDELINES

Sound exposure criteria essentially define those levels of sound from different sources that are likely to affect aquatic animals adversely, in order to regulate the generation of noise in aquatic environments. Significant efforts have been made over the past few years to develop criteria for aquatic vertebrates, including marine mammals, as well as guidelines for the use of these criteria (NMFS, 2018; Southall *et al.,* 2007).

Substantially less effort has been placed on developing criteria and guidelines for fishes. However, interim sound exposure criteria for the onset of physiological effects on fishes for use on the United States west coast were proposed by the Fisheries Hydroacoustics Working Group (FHWG, 2008) but also see Popper *et al.* (2006) and Woodbury and Stadler (2008). More recently, a new set of interim criteria was proposed (Popper *et al.,* 2014) based on a much stronger set of research and these raised the effective onset of effects levels, at least for physical effects, substantially and these interim criteria are now being used world‐wide (Andersson *et al.,* 2017).

Most work to date has focussed upon effects on marine mammals and marine fishes; much less is known about these effects in fresh water. However, Mickle and Higgs (2018) have recently reviewed the literature regarding behavioural and physiological effects of noise pollution on freshwater fish and have emphasised the lack of incorporation of both behavioural and physiological measures within current studies. Marine and freshwater soundscapes differ quite markedly and the transmission of sound through shallow lakes and rivers differs substantially from that under open‐water conditions in the sea. Substrate transmission of sound may be especially important in shallow freshwater environments. Thus, there is a need to examine those types and levels of sounds that are harmful to freshwater fishes and to establish relevant sound exposure criteria.

### 11.1 Current interim guidelines

The term onset and the phrase onset of effect have been widely used in preparing guidelines on sound exposure criteria. However, it is clear that onset is viewed very differently by different investigators, regulators and others and that there is no clear definition of the term, particularly with regard to the potential effects of sound on fishes. In this review, onset refers to the lowest sound level that results in a statistically significant effect, in terms of physical damage to an animal or a significant change in behaviour. It should be noted that earlier papers that considered fishes used onset for any level of response, including a response by a single animal in a school (Woodbury & Stadler, 2008). Thus, if there is scale loss in one fish within a group of many animals, that would be considered onset.

#### 11.1.1 Onset of physical effects

The interim sound exposure criteria, which are still in use, at least on the U.S. west coast (Caltrans, 2015; http://www.go.umd.edu/UcP), were based on a recommendation of dual criteria of peak sound pressure (SPL_peak_) and cumulative SEL (SEL_cum_) (Carlson *et al.,* 2007; Popper *et al.,* 2006; Popper & Hastings, 2009).

The rationale for dual criteria was that it was sometimes hard to determine one or the other measure when trying to set a signal level for onset of an effect and having alternative approaches provides a more conservative guideline for the protection of the animals. The SEL_cum_ was suggested since animals are often exposed to many more than a single pile driving strike in succession and any effect would probably come from an accumulation of energy from the multiple strikes. However, as noted above, it is now clear that the SEL_cum_ is probably an inappropriate measure of potential effects.

In 2008, the Fisheries Hydroacoustic Working Group adopted the interim dual‐criteria model for onset of physiological effects from sound exposure (FHWG, 2008). However, these criteria were immediately criticised since they were based on very limited scientific research on effects of pile driving on fishes (Carlson *et al.,* 2007; Popper & Hastings, 2009). The criteria were: Peak (SPL): 206 decibels (dB) re 1 μPa; SEL_cum_: 187 dB re 1 μPa^2^ s^−1^ for fishes above 2 g; SEL_cum_: 183 dB re 1 μPa^2^ s^−1^ for fishes below 2 g.

#### 11.1.2 Onset of behavioural effects

The U.S. National Marine Fisheries Service (NMFS), as well as other agencies, currently uses 150 dB re 1 μPa (rms) as the sound pressure level that may result in onset of behavioural effects (Caltrans, 2015). This is based on a recent NMFS guidance document (http://www.go.umd.edu/Ucs) that says that sound pressure above the 150 dB_rms_ level are expected to cause temporary changes in behaviour and these might include startle responses (though startle is not defined and has broad meaning to fish biologists), feeding disruption, area avoidance, *etc*. However, there are a number of problems with the 150 dB_rms_ criterion. First, its origin and scientific basis is not known (Hastings, 2008). Second, the value is based on the assumption that fishes respond to sound pressure even though, as pointed out earlier, most fishes primarily detect particle motion (see also Popper & Hawkins, 2018). Thus, any behavioural criteria should be based on the acoustic signals that the fish can actually detect and respond to. Finally, and perhaps most importantly, a single criterion value for behaviour does not take into consideration the very substantial species differences in hearing sensitivity, behaviour, *etc*., nor does it take into consideration response changes with animal age, season, or even motivational state (Neo *et al.,* 2014).

### 11.2 Recent criteria and guidelines

More recently, a set of interim criteria and guidelines for fishes was developed based on recent scientific advances (Table 2; Popper *et al.,* 2014). Of major importance, the authors concluded that it was not possible to define sound exposure criteria for every possible sound source, type of response to the sound, or do an analysis for every fish species (or even all of those potentially listed in various locales). Instead, they developed an approach that focussed on fish groups based on morphology of auditory apparatus (Table 3), on major sound types (*e.g*., pile driving, shipping) and major potential effects (Table 1). The overall intent was to provide the first science‐based, but clearly interim, criteria for effects of anthropogenic sound on fishes and to provide a way to deal with the potentially insurmountable combinations of species and sources. The authors very carefully, however, pointed out that the proposed criteria were not complete due to lack of data (Table 2 provides examples of the several effects tables found in the guidelines) and that they expected that as more studies were done, the suggested criteria would evolve.

**Table 3:** Grouping of Fishes as per 2014 Guidelines

Group	Characteristics
1	Fishes lacking swim bladders that are sensitive only to sound particle motion and show sensitivity to only a narrow band of frequencies (*e.g*., flatfishes, Pleuronectiformes; sharks skates and rays, Chondrichthyes).
2	Fishes with a swimbladder where that organ does not appear to play a role in hearing. These fish are sensitive only to particle motion and show sensitivity to only a narrow band of frequencies. This group includes salmonids (Salmonidae) and some tunas and mackerels (Scombridae), but many other species are likely to fit into this category as well.
3	Fishes with swim bladders that are close, but not intimately connected, to the ear. These fishes are sensitive to both particle motion and sound pressure, and show a more extended frequency range than groups 1 or 2, extending up to about 500 Hz. This group includes cod fishes (Gadidae), eels (Anguillidae), some drums and croakers (Sciaenidae), and perhaps other fishes.
4	Fishes that have special structures mechanically linking the swim bladder to the ear. These fishes are primarily sensitive to sound pressure, although they also detect particle motion. They have a wider frequency range, extending to several kHz and generally show higher sensitivity to sound pressure than fishes in groups 1, 2, or 3. The group includes some of the squirrelfishes (Holocentridae), drums and croakers (Sciaenidae), herrings (Clupeidae) and the large group of otophysan fishes.
5	Eggs and larvae.

Finally, the authors of the guidelines made it clear that many of the acoustic impact assessments carried out on fishes in the past and upon which the interim guidelines were based, must be amended since they only considered sound pressure and did not take into consideration the potential effects from high levels of particle motion, something that must be done in future iterations of the guidelines (Hawkins *et al.,* 2015; Hawkins & Popper, 2016b; Nedelec *et al.,* 2016; Popper & Hawkins, 2018). There is growing international awareness that fishes do possess particle‐motion receptors and that this must be taken into account in setting future criteria, once appropriate data are available.

### 11.3 European guidelines for fishes

The monitoring of underwater noise is included in the European Union's Marine Strategy Framework Directive (MSFD; EU, 2008), which is concerned with ensuring good environmental status (GES) of European waters (Andersson *et al.,* 2017; Dekeling *et al.,* 2016; Tasker *et al.,* 2010; Tasker *et al.,* 2012; van der Graaf *et al.,* 2012). The directive requires that the introduction of energy, including underwater noise, must be at levels that do not adversely affect the marine environment. No specific criteria for fishes are provided but indicators for achieving GES are specified.

The Swedish Environmental Protection Agency recently published a review that discusses regulation of pile driving sounds (Andersson *et al.,* 2017). While the review focusses on sound pressure, the authors also strongly concurred with the idea that future guidelines for fishes must also be in terms of particle motion and must also consider signals from the substrate. The proposed exposure values in the document were taken from the Popper *et al.* (2014) guidelines and follow the interim U.S. criteria. The sound pressure levels at which fish are at risk of death or sustaining serious injury to internal organs are considered to be SPL_peak_ 207 dB re 1 μPa, SEL_ss_ 174 dB re 1 μPa^2^ s^−1^ and SEL_cum_ 204 dB re 1 μPa^2^ s^−1^. Thresholds for fish larvae and eggs were based on the fact that no negative effects were observed at exposures of up to SPL_peak_ 217 dB re 1 μPa, SEL_ss_ 187 dB re 1 μPa^2^ s^−1^ and SEL_cum_ 207 dB re 1 μPa^2^ s^−1^. However, the paper notes that there are relatively few studies on the early life stages of fish. The Swedish review does not propose noise levels for flight behaviour or a temporary threshold shift (TTS) in fish because, unlike damage to internal organs, both flight behaviour and hearing damage are linked to the species’ specific sensitivity to frequency and sound intensity. And using the existing literature, it is not possible to assess whether flight behaviour negatively affects the species at the population level or whether the effect is related to the area and period of time.

In the UK, Nedwell *et al.* (2007) proposed a set of guidelines for behavioural responses utilising what they referred to as the dB_ht_ (species) concept. Nedwell *et al.* (2007) suggested that specific dB_ht_ levels above the hearing threshold of a fish elicited particular responses. The dB_ht_ is based on a frequency weighting approach since animals do not hear equally well at all frequencies within their hearing range. Frequency weighting is therefore often applied in assessing the effects of sounds upon particular species (*e.g*., Houser *et al.,* 2017). Weighting takes account of hearing ability by referencing sound levels to the species’ hearing thresholds. The Nedwell approach has been utilised within the UK for assessing the effects of anthropogenic sounds on fishes and it appears to have the tacit approval of some UK regulatory agencies. However, the dB_ht_ approach has very serious flaws that make it totally unacceptable (Hawkins & Popper, 2014, 2016b). This is suggested since Nedwell *et al.* (2007) concluded that strong avoidance responses by fishes start at a level about 90 dB above the dB_ht_ (species) thresholds, while different proportions of fishes respond at lower weighted levels. However, there are very few field data derived from wild fishes to support these chosen levels. Also, the concept of dB_ht_ has not been accepted in any independent peer‐reviewed publications. Indeed, extreme caution must be exercised in applying the dB_ht_ (species) measure. Defining response criteria applicable to all species is a far too simplistic an approach to evaluating behaviour. Moreover, the data on hearing thresholds used for the dB_ht_ approach should ideally be based on accurate behavioural threshold determinations rather than measures of inner ear responses, as the latter are susceptible to flaws (Sisneros *et al.,* 2016).

## 12 MAJOR RESEARCH GAPS AND RESEARCH RECOMMENDATIONS

In order to develop better guidelines and criteria, it will be critical to fill many gaps in understanding of the potential effects of anthropogenic sounds on fishes. The goal must be to increase knowledge in those areas that are most likely to enable sound exposure criteria to be revised, as per the expectation of the 2014 guidelines (Popper *et al.,* 2014). There are many research gaps suggested earlier in this paper and in other publications (Normandeau, 2012a,b; Hawkins *et al.,* 2015). Here we will focus on those questions and data needed to move forward most rapidly.

### 12.1 Selection of species

It is clear, based on the diversity of fishes and their life styles that it is critical to obtain data from multiple species and a range of sizes and ages of fish within each species. There is likely to be substantial variation in potential effects depending on differences in anatomy, physiology and behavioural responses to various stimuli. Recent guidelines (Popper *et al.,* 2014) suggested dividing fishes into several morphological groups that relate to the presence or absence and configuration of the swimbladder (see Tables 2 and 3). Having a representative set of species or fish types will be critical for future work on all aspects of effects of anthropogenic sound on fishes.

### 12.2 Behavioural responses

There is general consensus that the single most important issue is the effects of anthropogenic sound on fish behaviour. While questions about physical and physiological effects are important, the distance around the source that includes sounds of sufficient level to physically harm the fish is relatively small compared with the much greater area that is potentially ensonified, where the sounds are heard by the fish and where behavioural responses may be shown. Far fewer animals are likely to be directly harmed by sounds compared with the number of animals that may show changes in behaviour. Any anthropogenic sounds that alter the ability of animals to hear natural sounds that are important to them (*e.g*., as a result of masking), or cause temporary loss of hearing sensitivity (TTS), may affect their natural behaviour adversely. Some anthropogenic sounds may frighten the fish away from preferred locales or from migration routes. While many behavioural effects are likely to be minimal and have little or no effect on fish fitness and survival, some behavioural responses may have substantial short and long‐term effects upon them.

The currently available data on behavioural responses, as shown earlier, are highly variable and have many problems that do not even start to provide any general principles on how fishes respond to anthropogenic sound. Moreover, there are numerous additional behavioural issues that need to be examined, from the sound levels that are likely to influence hearing (*e.g*., hearing studies, studies of hearing in the presence of maskers) to responses to sound pressure *v*. responses to particle motion (Popper & Hawkins, 2018). Data are needed on general behavioural responses to sounds at different sound levels and how these responses change over time after the introduction of an anthropogenic source, as fishes may habituate to the sounds or temporarily show hearing losses due to the presence of persistent sounds. Especially significant is what fishes do when they are exposed to a particularly intense sound (do they move away or stay in place) and what are the long‐term consequences for fish populations?

Most importantly, long‐term, realistic field studies are needed on the effects of anthropogenic sounds on the behaviour of fishes, taking account of cumulative and synergistic effects, along with stress indicators. It is important to carry out such studies in the wild, where there are no constraints like tank walls or netting and where the acoustics are normal.

### 12.3 Effects of particle motion

It is now clear that fishes are primarily detectors of particle motion and relatively fewer species of fish use sound pressure. Thus, criteria and guidelines must be developed in terms of particle motion as well as sound pressure. Yet, very little is known about hearing sensitivity to particle motion and it is imperative that such data be obtained. Concurrently, it is imperative to measure the signal from anthropogenic sources in terms not only of sound pressure, as now done, but also in terms of particle motion.

### 12.4 Development of dose–response data

Studies on physical effects of pile driving signals in fishes are needed that could lead to understanding dose–response relationships of different sound variables such as signal intensity, number of strikes, inter‐strike interval, *etc*. Indeed, a recent study (Casper *et al.,* 2017) suggests that the dose–response relationship is more complex than previously thought. Studies of dose–response relationships will provide insight not only for understanding the onset of physical effects or behavioural effects, but also for determining those levels above the onset level at which potentially harmful effects start to occur. Such information will enable regulators and others to be able to make better decisions on criteria, particularly if they are willing to accept the idea that just because there is a small effect, this may not affect the fitness of the animal.

### 12.5 Hearing

Though here is a body of literature on the hearing of perhaps 100 fish species (Ladich & Fay, 2013), the greater portion of these data were obtained using sound pressure measures and do not reflect the fact that most fishes primarily detect particle motion. Moreover, most of the studies (particularly recently) used physiological measures (most often auditory evoked potentials; AEP) that do not reflect the sound processing capabilities of the whole auditory system and thus, do not reflect the actual hearing capabilities of an animal (Sisneros & Rogers, 2016). In order to understand fish hearing and the sounds that potential will affect behaviour, future studies must include particle motion and be done using behavioural methods that reflect how fishes actually respond to sound. Moreover, future studies need to be done in acoustic environments where sounds can be fully calibrated, such as in open bodies of water without physical constraints to reflect sounds, or in specially designed (and very expensive) tanks.

### 12.6 Population studies

In contrast with marine mammals, where populations are small and there is concern for single animals, the greater interest for fishes is with populations of animals. Loss of an individual due to exposure to anthropogenic sound does not have the same implications for a species as does the effect on a population. Effects are the broad range of potentially measurable changes that may be observed in individuals, groups of animals, or even habitats as a result of sound exposure. Impacts are effects that, with some certainty, rise to the level of deleterious ecological significance (Boehlert & Gill, 2010). Thus, the effect does not indicate the significance, whereas the impact deals with the severity, intensity, or duration of the effect upon animal populations and ecological communities. Such impacts can then be compared with those resulting from other stressors, including chemical pollution, fishing, pathogens, climate change *etc*. The ecosystem‐wide consequences of exposure to sound also need to be evaluated. Effects may influence the dynamics of predation and other types of biotic interactions at the community level. Making assessments across species and communities and within the wider ecosystem, may be of considerable value.

## 13 CONCLUSIONS

There is increasing concern about the effects of anthropogenic sounds upon aquatic animals, including fishes. It is evident, however, that there are major gaps in our understanding of the effects of these sounds and especially their effect upon animal populations and aquatic ecosystems. Much of the literature is limited in quality and many of the experiments have been carried out on captive fishes under laboratory conditions, rather than on free‐living fishes in the wild. There is also a lack of information on the responses to particle motion, rather than sound pressure. It is evident that there are so many information gaps that it is almost impossible to come to clear conclusions on the nature and levels of anthropogenic sound that have potential to cause changes in animal behaviour, or even physical harm. There is need to carry out further research on the behavioural responses of a range of fishes to different sound sources, under different conditions. As well as investigating responses to sounds of short duration, information is also required on responses to continuous or repeated exposure. What are the immediate effects of sound exposure and what are the longer‐term effects in terms of fitness and likely effect on populations?

At the same time, since there is an immediate need for updated criteria and guidelines on potential effects of anthropogenic sound on fishes, we recommend, as do our colleagues in Sweden (Andersson *et al.,* 2017), that the criteria proposed by Popper *et al.* (2014) should be used. (We recognise that the suggestion of using the 2014 guidelines is potentially self‐serving since we are lead authors on that document. However, as this document is growing in acceptance, we feel it important that we share our own thoughts and that of colleagues world‐wide.) However, as new data become available, these criteria need to be updated and filled in. We also suggest that there is significant need to define what onset of effect means in terms of fishes. Is this, as often now used, the start of any effect even on a single animal, or is it some level that, while easily assessed, reflects some statistical value and which focusses on the population rather than on individuals.

## CONTRIBUTIONS

A.N.P. and A.D.H. contributed equally to all aspects of this review.

## References

[jfb13948-bib-0001] Amorim, M. C. P. , Vasconcelos, R. O. , Bolgan, M. , Pedroso, S. S. , & Fonseca, P. J. (2018). Acoustic communication in marine shallow waters: Testing the acoustic adaptive hypothesis in sand gobies. The Journal of Experimental Biology, 221, jeb183681. 30171096 10.1242/jeb.183681

[jfb13948-bib-0002] Amoser, S. , Wysocki, L. E. , & Ladich, F. (2004). Noise emission during the first powerboat race in an alpine lake and potential impact on fish communities. The Journal of the Acoustical Society of America, 116, 3789–3797. 15658729 10.1121/1.1808219

[jfb13948-bib-0003] Andersson, M. H. , Andersson, S. , Ahlsen, J. , Andersoson, B. L. , Hammar, J. , Persson, L. K. , Pihl, J. , Sigray, P. & Wisstrom, A. (2017). A framework for regulating underwater noise during pile driving. A technical Vindal report. Stockholm: Environmental Protection agency, Stockholm, Sweden. http://www.tethys.pnnl.gov/sites/default/files/publications/Andersson-et-al-2017-Report6775.pdf

[jfb13948-bib-0004] Bass, A. H. , & Ladich, F. (2008). Vocal‐acoustic communication: From neurons to brain In Webb J. F., Fay R. R., & Popper A. N. (Eds.), Fish bioacoustics (pp. 253–278). New York, NY: Springer Science+Business Media, LLC.

[jfb13948-bib-0005] Benhaïm, D. , Péan, S. , Lucas, G. , Blanc, N. , Chatain, B. , & Bégout, M.‐L. (2012). Early life behavioural differences in wild caught and domesticated sea bass (*Dicentrarchus labrax*). Applied Animal Behaviour Science, 141, 79–90.

[jfb13948-bib-0006] Bittencourt, L. , Carvalho, R. R. , Lailson‐Brito, J. , & Azevedo, A. F. (2014). Underwater noise pollution in a coastal tropical environment. Marine Pollution Bulletin, 83, 331–336. 24814251 10.1016/j.marpolbul.2014.04.026

[jfb13948-bib-0007] Bolgan, M. , Chorazyczewska, E. , Winfield, I. J. , Codarin, A. , Brien, J. , & Gammell, M. (2016). First observations of anthropogenic underwater noise in a large multi‐use lake. Journal of Limnology, 75, 644–651.

[jfb13948-bib-0008] Bregman, A. S. (1994). Auditory scene analysis: The perceptual organization of sound. Boston, MA: MIT Press.

[jfb13948-bib-0009] Buscaino, G. , Filiciotto, F. , Buffa, G. , Bellante, A. , Di Stefano, V. , Assenza, A. , … Mazzola, S. (2010). Impact of an acoustic stimulus on the motility and blood parameters of European sea bass (*Dicentrarchus labrax* L.) and gilthead sea bream (*Sparus aurata* L.). Marine Environmental Research, 69, 136–142. 19828191 10.1016/j.marenvres.2009.09.004

[jfb13948-bib-0010] California Department of Transportation (2001). Pile installation demonstration project, fisheries impact assessment. In *San Francisco ‐ Oakland Bay Bridge East Span Seismic Safety Project*.

[jfb13948-bib-0011] Caltrans (2015). Technical guidance for assessment and mitigation of the hydroacoustics effects of pile driving on fish. p. 532. Sacramento, CA.

[jfb13948-bib-0012] Carlson, T. J. , Hastings, M. C. & Popper, A. N. (2007). Update on recommendations for revised interim sound exposure criteria for fish during pile driving activities. http://www.dot.ca.gov/hq/env/bio/files/ct-arlington_memo_12-21-07.pdf.

[jfb13948-bib-0013] Casaretto, L. , Picciulin, M. , & Hawkins, A. D. (2015). Seasonal patterns and individual differences in the calls of male haddock *Melanogrammus aeglefinus* . Journal of Fish Biology, 87, 579–603. 26333138 10.1111/jfb.12740

[jfb13948-bib-0014] Casper, B. M. , Carlson, T. J. , Halvorsen, M. B. , & Popper, A. N. (2016). Effects of impulsive pile‐driving exposure on fishes In Popper A. N. & Hawkins A. D. (Eds.), The effects of noise on aquatic life II (pp. 125–132). New York, NY: Springer. 10.1007/978-1-4939-2981-8_15 26610952

[jfb13948-bib-0015] Casper, B. M. , Halvorsen, M. B. , Carlson, T. J. , & Popper, A. N. (2017). Onset of barotrauma injuries related to number of pile driving strike exposures in hybrid striped bass. The Journal of the Acoustical Society of America, 141, 4380–4387. 28618820 10.1121/1.4984976

[jfb13948-bib-0016] Casper, B. M. , Halvorsen, M. B. , Matthews, F. , Carlson, T. J. , & Popper, A. N. (2013a). Recovery of barotrauma injuries resulting from exposure to pile driving sound in two sizes of hybrid striped bass. PLoS One, 8, e73844. 24040089 10.1371/journal.pone.0073844 PMC3770664

[jfb13948-bib-0017] Casper, B. M. , Popper, A. N. , Matthews, F. , Carlson, T. J. , & Halvorsen, M. B. (2012). Recovery of barotrauma injuries in Chinook salmon, *Oncorhynchus tshawytscha* from exposure to pile driving sound. PLoS One, 7, e39593. 22745794 10.1371/journal.pone.0039593 PMC3382140

[jfb13948-bib-0018] Casper, B. M. , Smith, M. E. , Halvorsen, M. B. , Sun, H. , Carlson, T. J. , & Popper, A. N. (2013b). Effects of exposure to pile driving sounds on fish inner ear tissues. Comparative Biochemistry and Physiology Part A: Molecular & Integrative Physiology, 166, 352–360. 10.1016/j.cbpa.2013.07.008 23850719

[jfb13948-bib-0019] Celi, M. , Filiciotto, F. , Maricchiolo, G. , Genovese, L. , Quinci, E. M. , Maccarrone, V. , … Buscaino, G. (2016). Vessel noise pollution as a human threat to fish: Assessment of the stress response in gilthead sea bream (*Sparus aurata*, Linnaeus 1758). Fish Physiology and Biochemistry, 42, 631–641. 26581747 10.1007/s10695-015-0165-3

[jfb13948-bib-0020] Chapman, C. , & Sand, O. (1974). Field studies of hearing in two species of flatfish *Pleuronectes platessa* (L.) and *Limanda limanda* (L.) (Family Pleuronectidae). Comparative Biochemistry and Physiology A, 47, 371–385. 10.1016/0300-9629(74)90082-6 4149016

[jfb13948-bib-0021] Chapman, C. J. , & Hawkins, A. (1973). A field study of hearing in the cod, *Gadus morhua* L. Journal of Comparative Physiology, 85, 147–167.

[jfb13948-bib-0022] Cheesman, S. (2016). Measurements of operational wind turbine noise in UKwaters In Popper A. N. & Hawkins A. D. (Eds.), The effects of noise on aquatic life II (pp. 153–160). New York, NY: Springer. 10.1007/978-1-4939-2981-8_18 26610955

[jfb13948-bib-0023] Dahl, P. H. , De Jong, C. , & Popper, A. N. (2015). The underwater sound field from impact pile driving and its potential effects on marine life. Acoustics Today, 11, 18–25.

[jfb13948-bib-0024] de Jong, C. A. , Ainslie, M. A. , Heinis, F. , & Janmaat, J. (2016). Offshore dredger sounds: Source levels, sound maps and risk assessment In Popper A. N. & Hawkins A. D. (Eds.), The effects of noise on aquatic life II (pp. 189–196). New York, NY: Springer. 10.1007/978-1-4939-2981-8_22 26610959

[jfb13948-bib-0025] de Jong, K. , Amorim, M. C. P. , Fonseca, P. J. , Fox, C. J. , & Heubel, K. U. (2017). Noise can affect acoustic communication and subsequent spawning success in fish. Environmental Pollution, 237, 814–823. 29146199 10.1016/j.envpol.2017.11.003

[jfb13948-bib-0026] De Robertis, A. , & Handegard, N. O. (2013). Fish avoidance of research vessels and the efficacy of noise‐reduced vessels: A review. ICES Journal of Marine Science, 70, 34–45.

[jfb13948-bib-0027] Dekeling, R. , Tasker, M. , Ainslie, M. , Andersson, M. , André, M. , Borsani, F. , … Folegot, T. (2016). The European marine strategy: Noise monitoring in European marine waters from 2014 In Popper A. N. & Hawkins A. D. (Eds.), The effects of noise on aquatic life II (pp. 205–215). New York, NY: Springer. 10.1007/978-1-4939-2981-8_24 26610961

[jfb13948-bib-0028] Duncan, A. J. , Lucke, K. , Erbe, C. , & McCauley, R. D. (2016). Issues associated with sound exposure experiments in tanks. Proceedings of Meetings on Acoustics, 27, 070008.

[jfb13948-bib-0029] El Balaa, R. , & Blouin‐Demers, G. (2011). Anti‐predatory behaviour of wild‐caught vs captive‐bred freshwater angelfish, *Pterophyllum scalare* . Journal of Applied Ichthyology, 27, 1052–1056.

[jfb13948-bib-0030] Ellison, W. T. , Southall, B. L. , Clark, C. W. , & Frankel, A. S. (2012). A new context‐based approach to assess marine mammal behavioral responses to anthropogenic sounds. Conservation Biology, 26, 21–28. 22182143 10.1111/j.1523-1739.2011.01803.x

[jfb13948-bib-0031] Engås, A. , & Lokkeborg, S. (2002). Effects of seismic shooting and vessel‐generated noise on fish behaviour and catch rates. Bioacoustics, 2, 313–316.

[jfb13948-bib-0032] Engås, A. , Løkkeborg, S. , Ona, E. , & Soldal, A. V. (1996). Effects of seismic shooting on local abundance and catch rates of cod (*Gadus morhua*) and haddock (*Melanogrammus aeglefinus*). Canadian Journal of Fisheries and Aquatic Sciences, 53, 2238–2249.

[jfb13948-bib-0033] Enger, P. S. (1967). Hearing in herring. Comparative Biochemistry and Physiology, 22, 527–538. 6075148 10.1016/0010-406x(67)90615-9

[jfb13948-bib-0034] Enger, P. S. (1981). Frequency discrimination in teleosts—Central or peripheral? In Tavolga W. A., Popper A. N., & Fay R. R. (Eds.), Hearing and sound communication in fishes (pp. 243–255). New York, NY: Springer.

[jfb13948-bib-0035] Erbe, C. (2013). Underwater noise of small personal watercraft (jet skis). The Journal of the Acoustical Society of America, 133, EL326–EL330. 23556699 10.1121/1.4795220

[jfb13948-bib-0036] Erbe, C. , MacGillivray, A. , & Williams, R. (2012). Mapping cumulative noise from shipping to inform marine spatial planning. The Journal of the Acoustical Society of America, 132, EL423–EL438. 23145705 10.1121/1.4758779

[jfb13948-bib-0037] Erbe, C. , Reichmuth, C. , Cunningham, K. , Lucke, K. , & Dooling, R. (2016). Communication masking in marine mammals: A review and research strategy. Marine Pollution Bulletin, 103, 15–38. 26707982 10.1016/j.marpolbul.2015.12.007

[jfb13948-bib-0038] EU . (2008). Directive 2008/56/EC of the European Parliament and of the Council of 17 June 2008 establishing a framework for community action in the field of marine environmental policy (Marine Strategy Framework Directive). Official Journal of the European Union, L 164, 19–40 Available at http://www.eur-lex.europa.eu/LexUriServ/LexUriServ.do?uri=OJ:L:2008:164:0019:0040:EN:PDF.

[jfb13948-bib-0039] Fay, R. R. (2005). Sound source localization by fishes In Popper A. N. & Fay R. R. (Eds.), Sound source localization. New York, NY: Springer‐Verlag.

[jfb13948-bib-0040] Fay, R. R. (2009). Soundscapes and the sense of hearing of fishes. Integrative Zoology, 4, 26–32. 21392274 10.1111/j.1749-4877.2008.00132.x

[jfb13948-bib-0041] Fay, R. R. , & Megela Simmons, A. (1999). The sense of hearing in fishes and amphibians In Fay R. R. & Popper A. N. (Eds.), Comparative hearing: Fish and amphibians (pp. 269–318). New York, NY: Springer‐Verlag.

[jfb13948-bib-0042] Fay, R. R. , & Popper, A. N. (1974). Acoustic stimulation of the ear of the goldfish (*Carassius auratus*). Journal of Experimental Biology, 61, 243–260. 4411820 10.1242/jeb.61.1.243

[jfb13948-bib-0043] FHWG (2008). Memorandum, agreement in principle for interim criteria for injury to fish from pile driving activities NOAA's Fisheries Northwest and Southwest Regions, US Fish and Wildlife Service Regions 1 and 8, Fisheries Hydroacoustic Working Group California/Washington/Oregon Departments of Transportation, California Department of Fish and Game, US Federal Highway Administration.

[jfb13948-bib-0044] Filiciotto, F. , Cecchini, S. , Buscaino, G. , Maccarrone, V. , Piccione, G. , & Fazio, F. (2016). Impact of aquatic acoustic noise on oxidative status and some immune parameters in gilthead sea bream *Sparus aurata* (Linnaeus, 1758) juveniles. Aquaculture Research, 48, 1895–1903.

[jfb13948-bib-0045] Gisiner, R. (2016). Sound and marine seismic surveys. Acoustics Today, 12, 10–18.

[jfb13948-bib-0046] Gordon, T. A. C. , Harding, H. R. , Wong, K. E. , Merchant, N. D. , Meekan, M. G. , McCormick, M. I. , … Simpson, S. D. (2018). Habitat degradation negatively affects auditory settlement behavior of coral reef fishes. Proceedings of the National Academy of Sciences, 115, 5193–5198. 10.1073/pnas.1719291115 PMC5960293 29712839

[jfb13948-bib-0047] Gourévitch, B. , Edeline, J.‐M. , Occelli, F. , & Eggermont, J. J. (2014). Is the din really harmless? Long‐term effects of non‐traumatic noise on the adult auditory system. Nature Reviews Neuroscience, 15, 483–491. 24946762 10.1038/nrn3744

[jfb13948-bib-0048] Gray, M. D. , Rogers, P. H. , Popper, A. N. , Hawkins, A. D. , & Fay, R. R. (2016). “Large” tank acoustics: How big is big enough? In Popper A. N. & Hawkins A. D. (Eds.), The effects of noise on aquatic life II (pp. 363–369). New York, NY: Springer. 10.1007/978-1-4939-2981-8_43 26610980

[jfb13948-bib-0049] Halvorsen, M. B. , Casper, B. M. , Carlson, T. J. , Woodley, C. M. , & Popper, A. N. (2012a). Assessment of barotrauma injury and cumulative sound exposure level in salmon after exposure to impulsive sound In Popper A. N. & Hawkins A. D. (Eds.), The effects of noise on aquatic life (pp. 235–237). New York, NY: Springer. 10.1007/978-1-4419-7311-5_52 22278489

[jfb13948-bib-0050] Halvorsen, M. B. , Casper, B. M. , Matthews, F. , Carlson, T. J. , & Popper, A. N. (2012b). Effects of exposure to pile‐driving sounds on the lake sturgeon, Nile tilapia and hogchoker. Proceedings of the Royal Society B, 279, 4705–4714. 23055066 10.1098/rspb.2012.1544 PMC3497083

[jfb13948-bib-0051] Halvorsen, M. B. , Casper, B. M. , Woodley, C. M. , Carlson, T. J. , & Popper, A. N. (2012c). Threshold for onset of injury in Chinook salmon from exposure to impulsive pile driving sounds. PLoS One, 7, e38968. 22745695 10.1371/journal.pone.0038968 PMC3380060

[jfb13948-bib-0052] Halvorsen, M. B. , Zeddies, D. G. , Chicoine, D. , & Popper, A. N. (2013). Effects of low‐frequency naval sonar exposure on three species of fish. The Journal of the Acoustical Society of America, 134, EL205–EL210. 23927226 10.1121/1.4812818

[jfb13948-bib-0053] Halvorsen, M. B. , Zeddies, D. G. , Ellison, W. T. , Chicoine, D. R. , & Popper, A. N. (2012d). Effects of mid‐frequency active sonar on hearing in fish. The Journal of the Acoustical Society of America, 131, 599–607. 22280622 10.1121/1.3664082

[jfb13948-bib-0054] Harwood, J. , King, S. , Schick, R. , Donovan, C. & Booth, C . (2014). A Protocol for Implementing the Interim Population Consequences of Disturbance (PCoD) Approach: Quantifying and Assessing the Effects of UK Offshore Renewable Energy Developments on Marine Mammal Populations. Scottish Marine and Freshwater Science Vol 5 No 2. Edinburgh: Marine Scotland. http://www.data.marine.gov.scot/dataset/protocol-implementing-interim-population-consequences-disturbance-pcod-approach-quantifying

[jfb13948-bib-0055] Hastings, M. C. (2008). Coming to terms with the effects of ocean noise on marine animals. Acoustics Today, 4, 22–34.

[jfb13948-bib-0056] Hastings, M. C. , Popper, A. N. , Finneran, J. J. , & Lanford, P. J. (1996). Effects of low‐frequency underwater sound on hair cells of the inner ear and lateral line of the teleost fish *Astronotus ocellatus* . The Journal of the Acoustical Society of America, 99, 1759–1766. 8819864 10.1121/1.414699

[jfb13948-bib-0057] Hawkins, A. , MacLennan, D. , Urquhart, G. , & Robb, C. (1974). Tracking cod *Gadus morhua* L. in a Scottish sea loch. Journal of Fish Biology, 6, 225–236.

[jfb13948-bib-0058] Hawkins, A. , Popper, A. N. , & Wahlberg, M. (2008). Introduction: International conference on the effects of noise on aquatic life. Bioacoustics, 17, 1–3.

[jfb13948-bib-0059] Hawkins, A. D. (1993). Underwater sound and fish behaviour In Pitcher T. J. (Ed.), Behaviour of teleost fishes (pp. 114–153). London, UK: Chapman and Hall.

[jfb13948-bib-0060] Hawkins, A. D. (2014). Examining fish in the sea: A European perspective on fish hearing experiments In Popper A. N. & Fay R. R. (Eds.), Perspectives on auditory research (pp. 247–267). New York, NY: Springer.

[jfb13948-bib-0061] Hawkins, A. D. , & Chapman, C. J. (1966). Underwater sounds of the haddock, *Melanogrammus aeglifinus* . Journal of the Marine Biological Association of the United Kingdom, 46, 241–247.

[jfb13948-bib-0062] Hawkins, A. D. , & Johnstone, A. D. F. (1978). The hearing of the Atlantic salmon, *Salmo salar* . Journal of Fish Biology, 13, 655–673.

[jfb13948-bib-0063] Hawkins, A. D. , & MacLennan, D. N. (1976). An acoustic tank for hearing studies on fish In Schuijf A. & Hawkins A. D. (Eds.), Sound reception in fish (pp. 149–170). Amsterdam, the Netherlands: Elsevier.

[jfb13948-bib-0064] Hawkins, A. D. , & Myrberg, A. A., Jr. (1983). Hearing and sound communication underwater In Lewis B. (Ed.), Bioacoustics, a comparative approach (pp. 347–405). New York, NY: Academic Press.

[jfb13948-bib-0065] Hawkins, A. D. , Pembroke, A. , & Popper, A. (2015). Information gaps in understanding the effects of noise on fishes and invertebrates. Reviews in Fish Biology and Fisheries, 25, 39–64.

[jfb13948-bib-0066] Hawkins, A. D. , & Popper, A. (2014). Assessing the impacts of underwater sounds on fishes and other forms of marine life. Acoustics Today, 10, 30–41.

[jfb13948-bib-0067] Hawkins, A. D. , & Popper, A. N. (2016a). Developing sound exposure criteria for fishes In Popper A. N. & Hawkins A. D. (Eds.), The effects of noise on aquatic life II (pp. 431–439). New York, NY: Springer. 10.1007/978-1-4939-2981-8_51 26610988

[jfb13948-bib-0068] Hawkins, A. D. , & Popper, A. N. (2016b). A sound approach to assessing the impact of underwater noise on marine fishes and invertebrates. ICES Journal of Marine Science: Journal du Conseil, 74, 635–671.

[jfb13948-bib-0069] Hawkins, A. D. , & Popper, A. N. (2018). Directional hearing and sound source localization by fishes. The Journal of the Acoustical Society of America, 144, 3329–3350. 30599653 10.1121/1.5082306

[jfb13948-bib-0070] Hawkins, A. D. , Roberts, L. , & Cheesman, S. (2014). Responses of free‐living coastal pelagic fish to impulsive sounds. The Journal of the Acoustical Society of America, 135, 3101–3116. 24926505 10.1121/1.4870697

[jfb13948-bib-0071] Hazelwood, R. , Macey, P. , Robinson, S. , & Wang, L. (2018). Optimal transmission of interface vibration wavelets—a simulation of seabed seismic responses. Journal of Marine Science and Engineering, 6, 61.

[jfb13948-bib-0072] Hazelwood, R. A. , & Macey, P. C. (2016). Modeling water motion near seismic waves propagating across a graded seabed, as generated by man‐made impacts. Journal of Marine Science and Engineering, 4, 47–61.

[jfb13948-bib-0073] Henderson, D. , & Hamernik, R. P. (2012). The use of kurtosis measurement in the assessment of potential noise trauma In Le Prell C. G., Henderson D., Fay R. R., & Popper A. N. (Eds.), Noise‐induced hearing loss: Scientific advances (pp. 41–55). New York, NY: Springer New York.

[jfb13948-bib-0074] Herbert‐Read, J. E. , Kremer, L. , Bruintjes, R. , Radford, A. N. & Ioannou, C. C. (2017). Anthropogenic noise pollution from pile‐driving disrupts the structure and dynamics of fish shoals. *Proceedings of the Royal Society B* 284. 10.1098/rspb.2017.1627 PMC5627215 28954915

[jfb13948-bib-0075] Hildebrand, J. A. (2009). Anthropogenic and natural sources of ambient noise in the ocean. Marine Ecology Progress Series, 395, 5–20.

[jfb13948-bib-0076] Hobday, A. , Smith, A. , Stobutzki, I. , Bulman, C. , Daley, R. , Dambacher, J. , … Furlani, D. (2011). Ecological risk assessment for the effects of fishing. Fisheries Research, 108, 372–384.

[jfb13948-bib-0077] Holt, D. E. , & Johnston, C. E. (2014). Evidence of the Lombard effect in fishes. Behavioral Ecology, 25, 819–826.

[jfb13948-bib-0078] Holt, D. E. , & Johnston, C. E. (2015). Traffic noise masks acoustic signals of freshwater stream fish. Biological Conservation, 187, 27–33.

[jfb13948-bib-0079] Houser, D. S. , Yost, W. , Burkard, R. , Finneran, J. , Reichmuth, C. , & Muslow, J. (2017). A review of the history, development and application of auditory weighting functions in humans and marine mammals. The Journal of the Acoustical Society of America, 141, 1371–1413. 28372133 10.1121/1.4976086

[jfb13948-bib-0080] Iafrate, J. D. , Watwood, S. L. , Reyier, E. A. , Scheidt, D. M. , Dossot, G. A. , & Crocker, S. E. (2016). Effects of pile driving on the residency and movement of tagged reef fish. PLoS One, 11, e0163638. 27880786 10.1371/journal.pone.0163638 PMC5120787

[jfb13948-bib-0081] Kane, A. S. , Song, J. , Halvorsen, M. B. , Miller, D. L. , Salierno, J. D. , Wysocki, L. E. , … Popper, A. N. (2010). Exposure of fish to high‐intensity sonar does not induce acute pathology. Journal of Fish Biology, 76, 1825–1840. 20557634 10.1111/j.1095-8649.2010.02626.x

[jfb13948-bib-0082] Kaplan, M. , Mooney, T. , Partan, J. , & Solow, A. (2015). Coral reef species assemblages are associated with ambient soundscapes. Marine Ecology Progress Series, 533, 93–107.

[jfb13948-bib-0083] Kaplan, M. B. , Mooney, T. A. , Lammers, M. O. , & Zang, E. (2016). Temporal and spatial variability in vessel noise on tropical coral reefs. Proceedings of Meetings on Acoustics, 27, 005002.

[jfb13948-bib-0084] Kastelein, R. A. , Gransier, R. , Marijt, M. A. T. , & Hoek, L. (2015). Hearing frequency thresholds of harbor porpoises (*Phocoena phocoena*) temporarily affected by played back offshore pile driving sounds. The Journal of the Acoustical Society of America, 137, 556–564. 25697990 10.1121/1.4906261

[jfb13948-bib-0085] Kastelein, R. A. , Jennings, N. , Kommeren, A. , Helder‐Hoek, L. , & Schop, J. (2017). Acoustic dose‐behavioral response relationship in sea bass (*Dicentrarchus labrax*) exposed to playbacks of pile driving sounds. Marine Environmental Research, 130, 315–324. 28874258 10.1016/j.marenvres.2017.08.010

[jfb13948-bib-0086] Kight, C. R. , & Swaddle, J. P. (2011). How and why environmental noise impacts animals: An integrative, mechanistic review. Ecology Letters, 14, 1052–1061. 21806743 10.1111/j.1461-0248.2011.01664.x

[jfb13948-bib-0087] Knudsen, F. R. , Enger, P. S. , & Sand, O. (1992). Awareness reactions and avoidance responses to sound in juvenile Atlantic salmon, *Salmo salar* L. Journal of Fish Biology, 40, 523–534.

[jfb13948-bib-0088] Ladich, F. (2014). Diversity in hearing in fishes: Ecoacoustical, communicative and developmental constraints In Köppl C., Manley G. A., Popper A. N., & Fay R. R. (Eds.), Insights from comparative hearing research (pp. 289–321). New York, NY: Springer New York.

[jfb13948-bib-0089] Ladich, F. , & Fay, R. R. (2013). Auditory evoked potential audiometry in fish. Reviews in Fish Biology and Fisheries, 23, 317–364. 26366046 10.1007/s11160-012-9297-z PMC4560088

[jfb13948-bib-0090] Ladich, F. , & Schulz‐Mirbach, T. (2016). Diversity in fish auditory systems: One of the riddles of sensory biology. Frontiers in Ecology and Evolution, 4, 1–26. 10.3389/fevo.2016.00028

[jfb13948-bib-0091] Løkkeborg, S. , Ona, E. , Vold, A. , Salthaug, A. , & Jech, J. M. (2012). Sounds from seismic air guns: Gear‐and species‐specific effects on catch rates and fish distribution. Canadian Journal of Fisheries and Aquatic Sciences, 69, 1278–1291.

[jfb13948-bib-0092] Lossent, J. , Lejart, M. , Folegot, T. , Clorennec, D. , Di Iorio, L. , & Gervaise, C. (2018). Underwater operational noise level emitted by a tidal current turbine and its potential impact on marine fauna. Marine Pollution Bulletin, 131, 323–334. 29886954 10.1016/j.marpolbul.2018.03.024

[jfb13948-bib-0093] Lucke, K. , Popper, A. N. , Hawkins, A. D. , Akamatsu, T. , André, M. , Branstetter, B. K. , … Mooney, T. A. (2016). Auditory sensitivity in aquatic animals. The Journal of the Acoustical Society of America, 139, 3097–3101. 27369131 10.1121/1.4952711

[jfb13948-bib-0094] Luczkovich, J. J. , & Keusenkothen, M. A. (2008). Can longspine squirrelfish hear bottlenose dolphin? Bioacoustics, 17, 75–77.

[jfb13948-bib-0095] Lumsdon, A. E. , Artamonov, I. , Bruno, M. C. , Righetti, M. , Tockner, K. , Tonolla, D. , & Zarfl, C. (2018). Soundpeaking – Hydropeaking induced changes in river soundscapes. River Research and Applications, 34, 3–12.

[jfb13948-bib-0096] MacGillivray, A. , Austin, M. , & Hannay, D. (2004). Underwater sound level and velocity measurements from study of airgun noise impacts on Mackenzie River fish species. Victoria, BC, Canada: JASCO Research Ltd.

[jfb13948-bib-0097] Madsen, P. T. , Wahlberg, M. , Tougaard, J. , Lucke, K. , & Tyack, P. (2006). Wind turbine underwater noise and marine mammals: Implications of current knowledge and data needs. Marine Ecology Progress Series, 309, 279–295.

[jfb13948-bib-0098] Mann, D. A. , Higgs, D. M. , Tavolga, W. N. , Souza, M. J. , & Popper, A. N. (2001). Ultrasound detection by clupeiform fishes. The Journal of the Acoustical Society of America, 109, 3048–3054. 11425147 10.1121/1.1368406

[jfb13948-bib-0099] Mann, D. A. , Lu, Z. , Hastings, M. C. , & Popper, A. N. (1998). Detection of ultrasonic tones and simulated dolphin echolocation clicks by a teleost fish, the American shad (*Alosa sapidissima*). The Journal of the Acoustical Society of America, 104, 562–568. 9670546 10.1121/1.423255

[jfb13948-bib-0100] Martin, B. , Zeddies, D. G. , Gaudet, B. , & Richard, J. (2016). Evaluation of three sensor types for particle motion measurement In Popper A. N. & Hawkins A. D. (Eds.), The effects of noise on aquatic life II (pp. 679–686). New York, NY: Springer. 10.1007/978-1-4939-2981-8_82 26611019

[jfb13948-bib-0101] Mattsson, A. , Parkes, G. , & Hedgeland, D. (2012). Svein Vaage broadband air gun study. Advances in Experimental Medicine and Biology, 730, 469–471. 22278543 10.1007/978-1-4419-7311-5_106

[jfb13948-bib-0102] McCauley, R. D. , Fewtrell, J. , & Popper, A. N. (2003). High intensity anthropogenic sound damages fish ears. The Journal of the Acoustical Society of America, 113, 638–642. 12558299 10.1121/1.1527962

[jfb13948-bib-0103] Mickle, M. F. , & Higgs, D. M. (2018). Integrating techniques: A review of the effects of anthropogenic noise on freshwater fish. Canadian Journal of Fisheries and Aquatic Sciences, 75, 1535–1541.

[jfb13948-bib-0104] Mickle, M. F. , Miehls, S. M. , Johnson, N. S. , & Higgs, D. M. (2018). Hearing capabilities and behavioural response of sea lamprey (*Petromyzon marinus*) to low frequency sounds. Canadian Journal of Fisheries and Aquatic Sciences.

[jfb13948-bib-0105] Montgomery, J. C. , Jeffs, A. , Simpson, S. D. , Meekan, M. , & Tindle, C. (2006). Sound as an orientation cue for the pelagic larvae of reef fishes and decapod crustaceans. Advances in Marine Biology, 51, 143–196. 16905427 10.1016/S0065-2881(06)51003-X

[jfb13948-bib-0106] Moulton, J. M. (1963). Acoustic behaviour of fishes In Busnel R. G. (Ed.), Acoustic behaviour of animals (pp. 655–693). Amsterdam, the Netherlands: Elsevier.

[jfb13948-bib-0107] Nedelec, S. L. , Campbell, J. , Radford, A. N. , Simpson, S. D. , & Merchant, N. D. (2016). Particle motion: The missing link in underwater acoustic ecology. Methods in Ecology and Evolution, 7, 836–842.

[jfb13948-bib-0108] Nedelec, S. L. , Simpson, S. D. , Morley, E. L. , Nedelec, B. & Radford, A. N. (2015). Impacts of regular and random noise on the behaviour, growth and development of larval Atlantic cod (*Gadus morhua*). *Proceedings of the Royal Society B* **282**. 10.1098/rspb.2015.1943 PMC4633878 26468248

[jfb13948-bib-0109] Nedwell, J. R. , Turnpenny, A. W. H. , Lovell, J. , Parvin, S. J. , Workman, R. , J.A.L., S . & Howell, D. (2007). A validation of the dBht as a measure of the behavioural and auditory effects of underwater noise. Report by Subacoustech Ltd. p. 78.

[jfb13948-bib-0110] Neo, Y. Y. , Seitz, J. , Kastelein, R. A. , Winter, H. V. , ten Cate, C. , & Slabbekoorn, H. (2014). Temporal structure of sound affects behavioural recovery from noise impact in European seabass. Biological Conservation, 178, 65–73.

[jfb13948-bib-0111] NMFS (2018). 2018 Revisions to: Technical Guidance for Assessing the Effects of Anthropogenic Sound on Marine Mammal Hearing (Version 2.0): Underwater Thresholds for Onset of Permanent and Temporary Threshold Shifts. p. 167. Washington, DC: US Department of Commerce.

[jfb13948-bib-0112] Normandeau (2012a). Effects of noise on fish, fisheries and invertebrates in the U.S. Atlantic and Arctic from energy industry sound‐generating activities. A Literature Synthesis for the US Dept of the Interior, Bureau of Ocean Energy Management.

[jfb13948-bib-0113] Normandeau (2012b). Effects of noise on fish, fisheries and invertebrates in the US Atlantic and Arctic from energy industry sound‐generating activities. A Workshop Report for the US Dept of the Interior, Bureau of Ocean Energy Management.

[jfb13948-bib-0114] NRC . (1994). Low‐frequency sound and marine mammals: Current knowledge and research need. Washington, DC: National Research Council National Academy Press. 25144092

[jfb13948-bib-0115] NRC . (2005). Marine mammal populations and ocean noise: Determining when noise causes biologically significant effects. Washington, DC: National Research Council National Academy Press.

[jfb13948-bib-0116] Oldfield, R. G. (2011). Aggression and welfare in a common aquarium fish, the Midas cichlid. Journal of Applied Animal Welfare Science, 14, 340–360. 21932947 10.1080/10888705.2011.600664

[jfb13948-bib-0117] Ona, E. , Godoo/, O. R. , Handegard, N. O. , Hjellvik, V. , Patel, R. , & Pedersen, G. (2007). Silent research vessels are not quiet. The Journal of the Acoustical Society of America, 121, EL145–EL150. 17471759 10.1121/1.2710741

[jfb13948-bib-0118] Pangerc, T. , Theobald, P. D. , Wang, L. S. , Robinson, S. P. , & Lepper, P. A. (2016). Measurement and characterisation of radiated underwater sound from a 3.6 MW monopile wind turbine. The Journal of the Acoustical Society of America, 140, 2913–2922. 27794307 10.1121/1.4964824

[jfb13948-bib-0119] Parvulescu, A. (1964). Problems of propagation and processing In Tavolga W. N. (Ed.), Marine bio‐acoustics (pp. 87–100). Oxford, UK: Pergamon Press.

[jfb13948-bib-0120] Patrick, W. S. , Spence, r. P. , Link, J. , Cope, J. , Field, J. , Kobayash, i. D. , … Overholtz, W. (2010). Using productivity and susceptibility indices to assess the vulnerability of United States fish stocks to overfishing. Fisheries Bulletin, 108, 305–322.

[jfb13948-bib-0121] Petersson, E. , Valencia, A. C. , & Järvi, T. (2015). Failure of predator conditioning: An experimental study of predator avoidance in brown trout (*Salmo trutta*). Ecology of Freshwater Fish, 24, 329–337.

[jfb13948-bib-0122] Pine, M. K. , Jeffs, A. G. , Wang, D. , & Radford, C. A. (2016). The potential for vessel noise to mask biologically important sounds within ecologically significant embayments. Ocean & Coastal Management, 127, 63–73.

[jfb13948-bib-0123] Pirotta, E. , Booth, C. G. , Costa, D. P. , Fleishman, E. , Kraus, S. D. , Lusseau, D. , … Harwood, J. (2018). Understanding the population consequences of disturbance. Ecology and Evolution, 8, 9934–9946. 30386587 10.1002/ece3.4458 PMC6202709

[jfb13948-bib-0124] Popper, A. N. , Carlson, T. J. , Hawkins, A. D. , Southall, B. L. & Gentry, R. L. (2006). Interim criteria for injury of fish exposed to pile driving operations: A white paper. In *Report to the Fisheries Hydroacoustic Working Group,* California Department of Transportation*,* USA*,* 15 pp.

[jfb13948-bib-0125] Popper, A. N. , & Coombs, S. (1982). The morphology and evolution of the ear in actinopterygian fishes. American Zoologist, 22, 311–328.

[jfb13948-bib-0126] Popper, A. N. , & Fay, R. R. (2011). Rethinking sound detection by fishes. Hearing Research, 273, 25–36. 20034550 10.1016/j.heares.2009.12.023

[jfb13948-bib-0127] Popper, A. N. , Fay, R. R. , Platt, C. , & Sand, O. (2003). Sound detection mechanisms and capabilities of teleost fishes In Collin S. P. & Marshall N. J. (Eds.), Sensory processing in aquatic environments (pp. 3–38). New York, NY: Springer‐Verlag.

[jfb13948-bib-0128] Popper, A. N. , Gross, J. A. , Carlson, T. J. , Skalski, J. , Young, J. V. , Hawkins, A. D. , & Zeddies, D. (2016). Effects of exposure to the sound from seismic airguns on pallid sturgeon and paddlefish. PLoS One, 11, e0159486. 27505029 10.1371/journal.pone.0159486 PMC4978428

[jfb13948-bib-0129] Popper, A. N. , Halvorsen, M. B. , Carlson, T. J. , Smith, M. E. , & Casper, B. M. (2013). Effects of pile driving on fishes. The Journal of the Acoustical Society of America, 134, 4059.

[jfb13948-bib-0130] Popper, A. N. , Halvorsen, M. B. , Kane, A. S. , Miller, D. L. , Smith, M. E. , Song, J. , … Wysocki, L. E. (2007). The effects of high‐intensity, low‐frequency active sonar on rainbow trout. The Journal of the Acoustical Society of America, 122, 623–635. 17614519 10.1121/1.2735115

[jfb13948-bib-0131] Popper, A. N. , & Hastings, M. C. (2009). The effects of anthropogenic sources of sound on fishes. Journal of Fish Biology, 75, 455–489. 20738551 10.1111/j.1095-8649.2009.02319.x

[jfb13948-bib-0132] Popper, A. N. , & Hawkins, A. D. (2012). The effects of noise on aquatic life. New York, NY: Springer Science+Business Media.

[jfb13948-bib-0133] Popper, A. N. , & Hawkins, A. D. (2016). The effects of noise on aquatic life, II. New York, NY: Springer Science+Business Media.

[jfb13948-bib-0134] Popper, A. N. , & Hawkins, A. D. (2018). The importance of particle motion to fishes and invertebrates. The Journal of the Acoustical Society of America, 143, 470–486. 29390747 10.1121/1.5021594

[jfb13948-bib-0135] Popper, A. N. , Hawkins, A. D. , Fay, R. R. , Mann, D. A. , Bartol, S. , Carlson, T. J. , … Tavolga, W. A. (2014). ASA S3 s^*−1*^C1. 4 TR‐2014 sound exposure guidelines for fishes and sea turtles: A technical report prepared by ANSI‐accredited standards committee S3 s^*−1*^C1 and registered with ANSI. New York, NY: Springer.

[jfb13948-bib-0136] Popper, A. N. , & Hoxter, B. (1987). Sensory and nonsensory ciliated cells in the ear of the sea lamprey, *Petromyzon marinus* . Brain, Behavior and Evolution, 30, 43–61. 10.1159/000118637 2887234

[jfb13948-bib-0137] Popper, A. N. , Smith, M. E. , Cott, P. A. , Hanna, B. W. , MacGillivray, A. O. , Austin, M. E. , & Mann, D. A. (2005). Effects of exposure to seismic airgun use on hearing of three fish species. The Journal of the Acoustical Society of America, 117, 3958–3971. 16018498 10.1121/1.1904386

[jfb13948-bib-0138] Purser, J. , & Radford, A. N. (2011). Acoustic noise induces attention shifts and reduces foraging performance in three‐spined sticklebacks (*Gasterosteus aculeatus*). PLoS One, 6, e17478. 21386909 10.1371/journal.pone.0017478 PMC3046255

[jfb13948-bib-0139] Putland, R. L. , Montgomery, J. C. , & Radford, C. A. (2018). Ecology of fish hearing. Journal of Fish Biology, 1–14. 10.1111/jfb.13867 30447064

[jfb13948-bib-0140] Radford, A. N. , Kerridge, E. , & Simpson, S. D. (2014). Acoustic communication in a noisy world: Can fish compete with anthropogenic noise? Behavioral Ecology, 25, 1022–1030.

[jfb13948-bib-0141] Radford, A. N. , Lèbre, L. , Lecaillon, G. , Nedelec, S. L. , & Simpson, S. D. (2016). Repeated exposure reduces the response to impulsive noise in European seabass. Global Change Biology, 22, 3349–3360. 27282635 10.1111/gcb.13352 PMC5006868

[jfb13948-bib-0142] Radford, C. A. , Stanley, J. A. , Tindle, C. T. , Montgomery, J. C. , & Jeffs, A. G. (2010). Localised coastal habitats have distinct underwater sound signatures. Marine Ecology Progress Series, 401, 21–29.

[jfb13948-bib-0143] Ramcharitar, J. , Gannon, D. P. , & Popper, A. N. (2006). Bioacoustics of the family Sciaenidae (croakers and drumfishes). Transactions of the American Fisheries Society, 135, 1409–1431.

[jfb13948-bib-0144] Remage‐Healey, L. , & Bass, A. H. (2006). From social behavior to neural circuitry: Steroid hormones rapidly modulate advertisement calling via a vocal pattern generator. Hormones and Behaviour, 50, 432–441. 10.1016/j.yhbeh.2006.05.007 16870192

[jfb13948-bib-0145] Retzius, G. (1881). Das Gehörorgan der Wirbelthiere. Stockholm: Samson and Wallin.

[jfb13948-bib-0146] Roberts, L. , & Breithaupt, T. (2016). Sensitivity of crustaceans to substrate‐borne vibration In Popper A. N. & Hawkins A. D. (Eds.), The effects of noise on aquatic life II (pp. 925–931). New York, NY: Springer. 10.1007/978-1-4939-2981-8_114 26611051

[jfb13948-bib-0147] Roberts, L. , Cheesman, S. , & Hawkins, A. D. (2016a). Effects of sound on the behavior of wild, unrestrained fish schools In Popper A. N. & Hawkins A. D. (Eds.), The effects of noise on aquatic life II (pp. 917–924). New York, NY: Springer. 10.1007/978-1-4939-2981-8_113 26611050

[jfb13948-bib-0148] Roberts, L. , Pérez‐Domínguez, R. , & Elliott, M. (2016b). Use of baited remote underwater video (BRUV) and motion analysis for studying the impacts of underwater noise upon free ranging fish and implications for marine energy management. Marine Pollution Bulletin, 112, 75–85. 27622927 10.1016/j.marpolbul.2016.08.039

[jfb13948-bib-0149] Rogers, P. H. , Hawkins, A. D. , Popper, A. N. , Fay, R. R. , & Gray, M. D. (2016). Parvulescu revisited: Small tank acoustics for bioacousticians In Popper A. N. & Hawkins A. D. (Eds.), The effects of noise on aquatic life, II (pp. 933–941). New York, NY: Springer Science+Business Media. 10.1007/978-1-4939-2981-8_115 26611052

[jfb13948-bib-0150] Ross, D. (1987). Mechanics of underwater noise. Los Altos, CA: Peninsula Publishing.

[jfb13948-bib-0151] Ross, D. (1993). On ocean underwater ambient noise. Acoustics Bulletin, 18, 5–8.

[jfb13948-bib-0152] Rossi, E. , Licitra, G. , Iacoponi, A. , & Taburni, D. (2016). Assessing the underwater ship noise levels in the North Tyrrhenian Sea In The effects of noise on aquatic life II (pp. 943–949). New York, NY: Springer. 10.1007/978-1-4939-2981-8_116 26611053

[jfb13948-bib-0153] Sand, O. , & Bleckmann, H. (2008). Orientation to auditory and lateral line stimuli In Webb J. F., Fay R. R., & Popper A. N. (Eds.), Fish bioacoustics (pp. 183–222). New York, NY: Springer Science+Business Media, LLC.

[jfb13948-bib-0154] Sand, O. , & Enger, P. S. (1973). Function of the swimblabber in fish hearing In Moller A. (Ed.), Basic mechanisms of hearing (pp. 893–908). New York, NY: Academic Press.

[jfb13948-bib-0155] Sand, O. , & Hawkins, A. D. (1973). Acoustic properties of the cod swim bladder. Journal of Experimental Biology, 58, 797–820.

[jfb13948-bib-0156] Sand, O. , & Karlsen, H. E. (2000). Detection of infrasound and linear acceleration in fishes. Philosophical Transactions of the Royal Society B, 355, 1295–1298. 10.1098/rstb.2000.0687 PMC1692823 11079418

[jfb13948-bib-0157] Schramm, M. P. , Bevelhimer, M. , & Scherelis, C. (2017). Effects of hydrokinetic turbine sound on the behavior of four species of fish within an experimental mesocosm. Fisheries Research, 190, 1–14.

[jfb13948-bib-0158] Schulz‐Mirbach, T. , Hess, M. , Metscher, B. D. , & Ladich, F. (2013). A unique swim bladder‐inner ear connection in a teleost fish revealed by a combined high‐resolution microtomographic and three‐dimensional histological study. BMC Biology, 11, 75. 23826967 10.1186/1741-7007-11-75 PMC3720219

[jfb13948-bib-0159] Schulz‐Mirbach, T. , & Ladich, F. (2016). Diversity of inner ears in fishes: Possible contribution towards hearing improvements and evolutionary considerations In Sisneros J. A. (Ed.), Fish hearing and bioacoustics: An anthology in honor of Arthur N. Popper & Richard R. Fay (pp. 341–391). Cham: Springer International Publishing. 10.1007/978-3-319-21059-9_16 26515322

[jfb13948-bib-0160] Schulz‐Mirbach, T. , Ladich, F. , Plath, M. , & BeB, M. (2018). Enigmatic ear stones: What we know about the functional role and evolution of fish otoliths. Biological Reviews, 457–482. 10.1111/brv.12463 30239135

[jfb13948-bib-0161] Sertlek, H. Ö. , Aarts, G. , Brasseur, S. , Slabbekoorn, H. , ten Cate, C. , von Benda‐Beckmann, A. M. , & Ainslie, M. A. (2016). Mapping underwater sound in the Dutch part of the North Sea In Popper A. N. & Hawkins A. D. (Eds.), The effects of noise on aquatic life II (pp. 1001–1006). New York, NY: Springer. 10.1007/978-1-4939-2981-8_124 26611061

[jfb13948-bib-0162] Shafiei Sabet, S. , Wesdorp, K. , Campbell, J. , Snelderwaard, P. , & Slabbekoorn, H. (2016). Behavioural responses to sound exposure in captivity by two fish species with different hearing ability. Animal Behaviour, 116, 1–11.

[jfb13948-bib-0163] Sierra‐Flores, R. , Atack, T. , Migaud, H. , & Davie, A. (2015). Stress response to anthropogenic noise in Atlantic cod *Gadus morhua* L. Aquacultural Engineering, 67, 67–76.

[jfb13948-bib-0164] Sigray, P. , & Andersson, M. H. (2012). Underwater particle acceleration induced by a wind turbine in the Baltic Sea In Popper A. N. & Hawkins A. D. (Eds.), The effects of noise on aquatic life (pp. 489–492). New York, NY: Springer. 10.1007/978-1-4419-7311-5_111 22278548

[jfb13948-bib-0165] Sisneros, J. A. , Popper, A. N. , Hawkins, A. D. , & Fay, R. R. (2016). Auditory evoked potential audiograms compared to behavioral audiograms in aquatic animals In Popper A. N. & Hawkins A. D. (Eds.), The effects of noise on aquatic life, II (pp. 1049–1056). New York, NY: Springer Science+Business Media. 10.1007/978-1-4939-2981-8_130 26611067

[jfb13948-bib-0166] Sisneros, J. A. , & Rogers, P. H. (2016). Directional hearing and sound source localization in fishes In Sisneros J. A. (Ed.), Fish hearing and bioacoustics: an anthology in honor of Arthur N. Popper & Richard R. Fay (pp. 121–155). Cham: Springer International Publishing. 10.1007/978-3-319-21059-9_7 26515313

[jfb13948-bib-0167] Slabbekoorn, H. (2018). Soundscape ecology of the Anthropocene. Acoustics Today, 14, 42–49.

[jfb13948-bib-0168] Slabbekoorn, H. , Dooling, R. J. , Popper, A. N. , & Fay, R. R. (2018). Effects of anthropogenic noise on animals. New York, NY: Springer.

[jfb13948-bib-0169] Smith, A. , Fulton, E. , Hobday, A. , Smith, D. , & Shoulder, P. (2007). Scientific tools to support the practical implementation of ecosystem‐based fisheries management. ICES Journal of Marine Science: Journal du Conseil, 64, 633–639.

[jfb13948-bib-0170] Smith, M. E. , Coffin, A. B. , Miller, D. L. , & Popper, A. N. (2006). Anatomical and functional recovery of the goldfish (*Carassius auratus*) ear following noise exposure. The Journal of Experimental Biology, 209, 4193–4202. 17050834 10.1242/jeb.02490

[jfb13948-bib-0171] Smith, M. E. , Kane, A. S. , & Popper, A. N. (2004). Acoustical stress and hearing sensitivity in fishes: Does the linear threshold shift hypothesis hold water? Journal of Experimental Biology, 207, 3591–3602. 15339955 10.1242/jeb.01188

[jfb13948-bib-0172] Smith, M. E. , & Monroe, J. D. (2016). Causes and consequences of sensory hair cell damage and recovery in fishes In Sisneros J. (Ed.), Fish hearing and bioacoustics (pp. 393–417). New York, NY: Springer. 10.1007/978-3-319-21059-9_17 26515323

[jfb13948-bib-0173] Smith, M. E. , Schuck, J. B. , Gilley, R. R. , & Rogers, B. D. (2011). Structural and functional effects of acoustic exposure in goldfish: Evidence for tonotopy in the teleost saccule. BMC Neuroscience, 12, 19. 21324138 10.1186/1471-2202-12-19 PMC3050771

[jfb13948-bib-0174] Southall, B. L. (2005). Final report of the 2004 international symposium “Shipping noise and marine mammals: A forum for science, technology and management.” In National marine fisheries service, office of protected resources, technical report. Washington, DC: National Oceanic and Atmospheric Administration.

[jfb13948-bib-0175] Southall, B. L. , Bowles, A. E. , Ellison, W. T. , Finneran, J. J. , Gentry, R. L. , Greene, C. R., Jr. , … Tyack, P. L. (2007). Marine mammal noise exposure criteria: Initial scientific recommendations. Aquatic Mammals, 33, 411–521.

[jfb13948-bib-0176] Spiga, I. , Aldred, N. , & Caldwell, G. S. (2017). Anthropogenic noise compromises the anti‐predator behaviour of the European seabass, *Dicentrarchus labrax* (L.). Marine Pollution Bulletin, 122, 297–305. 28662977 10.1016/j.marpolbul.2017.06.067

[jfb13948-bib-0177] Stanley, J. A. , Radford, C. A. , & Jeffs, A. G. (2012). Effects of underwater noise on larval settlement In Popper A. N. & Hawkins A. D. (Eds.), The effects of noise on aquatic life. New York, NY: Springer‐Verlag. 10.1007/978-1-4419-7311-5_84 22278521

[jfb13948-bib-0178] Tasker, M. , Amundin, M. , Andre, M. , Hawkins, A. , Lang, W. , Merck, T. , Scholik‐Schlomer, A. , Teilmann, J. , Thomsen, F. & Werner, S. (2010). Marine Stategy Framework Diretive Task Group 11 Report Underwater noise and other forms of energy. *Report No. EUR* 24341. 10.1007/978-1-4419-7311-5_132 22278569

[jfb13948-bib-0179] Tasker, M. , Amundin, M. , Andre, M. , Hawkins, A. D. , Lang, W. , Merck, T. , … Zakharia, M. (2012). Managing underwater noise in European waters: Implementing the marine strategy framework directive In Popper A. N. & Hawkins A. D. (Eds.), Effects of noise on aquatic life (pp. 583–585). New York, NY: Springer. 10.1007/978-1-4419-7311-5_132 22278569

[jfb13948-bib-0180] Tavolga, W. N. (1971). Sound production and detection In Hoar W. S. & Randall D. J. (Eds.), Fish physiology (pp. 135–205). New York, NY: Academic Press.

[jfb13948-bib-0181] Tennessen, J. B. , Parks, S. E. , & Langkilde, T. L. (2016). Anthropogenic noise and physiological stress in wildlife In Popper A. N. & Hawkins A. (Eds.), The effects of noise on aquatic life II (pp. 1145–1148). New York, NY: Springer. 10.1007/978-1-4939-2981-8_142 26611079

[jfb13948-bib-0182] Urick, R. J. (1983). Principles of underwater sound. New York, NY: McGraw‐Hill.

[jfb13948-bib-0183] van der Graaf, A. , Ainslie, M. , André, M. , Brensing, K. , Dalen, J. , Dekeling, R. , Robinson, S. , Tasker, M. , Thomsen, F. & Werner, S. (2012). European Marine Strategy Framework Directive‐Good Environmental Status (MSFD GES): Report of the Technical Subgroup on Underwater noise and other forms of energy. Brussels. http://www.iqoe.org/library/8061

[jfb13948-bib-0184] Webb, J. F. , Fay, R. R. , & Popper, A. N. (2008). Fish bioacoustics. New York, NY: Springer.

[jfb13948-bib-0185] Weilgart , L. (2017). The impact of ocean noise pollution on fish and invertebrates. Report for OceanCare, Switzerland. 23 pp.

[jfb13948-bib-0186] Wenger, A. S. , Harvey, E. , Wilson, S. , Rawson, C. , Newman, S. J. , Clarke, D. , … Evans, R. D. (2017). A critical analysis of the direct effects of dredging on fish. Fish and Fisheries, 18, 967–985.

[jfb13948-bib-0187] Woodbury, D. , & Stadler, J. (2008). A proposed method to assess physical injury to fishes from underwater sound produced during pile driving. Bioacoustics, 17, 289–297.

[jfb13948-bib-0188] Wysocki, L. E. , Dittami, J. P. , & Ladich, F. (2006). Ship noise and cortisol secretion in European freshwater fishes. Biological Conservation, 128, 501–508.

